# Euphemisms modulate the neural processing of norm violations

**DOI:** 10.3389/fnhum.2026.1917736

**Published:** 2026-09-23

**Authors:** Yulia Gorodnicheva, Eliana Monahhova, Amir Bekim, Qiang Shen, Vasily Klucharev

**Affiliations:** 1 Institute for Cognitive Neurosciences, National Research University Higher School of Economics, Moscow, Russia; 2 Shanghai International Studies University, Shanghai, China

**Keywords:** ERP, euphemisms, framing, moral evaluations, norm violations, P600

## Abstract

Euphemisms are commonly used in media to soften or obscure the severity of unethical or controversial actions. The present study explored the neural correlates underlying the processing of euphemized vignettes portraying norm violations. We recorded event-related potentials (ERPs) and collected behavioral severity ratings while participants (*n* = 38) read vignettes describing the same norm-violating events framed with euphemized, non-euphemized or control critical words. Behaviorally, participants rated norm violations as significantly less severe when described with euphemisms. At the neurophysiological level, non-euphemized critical words evoked significantly larger P600 amplitudes than euphemized words, whereas no reliable modulation was observed on the N400 component. Previous ERP studies have linked the N400 to the detection of semantic and normative incongruities, whereas the P600 has been associated with later integrative processes and evaluative reappraisal during language comprehension. These results suggest that euphemisms primarily modulate late-stage evaluative reappraisal and integration of norm violations, rather than early lexico-semantic detection.

## 1 Introduction

Norm-violation detection is an ancient feature of human social cognition and is tightly linked to neural and behavioral responses that support sanctioning and deterrence (Fehr and Fischbacher, 2004; Krueger and Hoffman, 2016; Eriksson et al., 2021). Fast detection of violating actions is crucial for reinforcing social norms and promoting consistent normative behavior (Richerson and Boyd, 2005; Cialdini and Goldstein, 2004). At the same time, normative evaluations are not determined solely by the underlying act: they can be shifted by how the same norm violation is described. In contemporary information environments, such linguistic framing is routinely leveraged as a media manipulation to justify immoral behavior or soften perceived norm violations (Susser et al., 2019). In the current study, we investigated the neural correlates of media framing that employs euphemisms to portray norm violations (euphemistic framing).

Euphemisms are widely used in political and media discourse to obscure controversial realities, frame ideology, and manage public perception (for a review, see Ahmed and Jomaa, 2022; Chilton, 2004; Crespo-Fernández, 2014). Euphemisms are typically defined as milder, more indirect, or more abstract expressions that replace more direct or emotionally loaded terms while referring to the same underlying events (Allan and Burridge, 2006; Crespo-Fernández, 2014; Leech, 1974; Linfoot-Ham, 2005). Unlike dysphemisms, which exaggerate negativity, or more general forms of doublespeak, euphemisms characteristically soften evaluations and emotional impact while preserving the core factual content. For example, “wounded civilian” (non-euphemism) could be reported as “collateral damage” (euphemism) to attenuate emotional responses to civilian casualties (Allan and Burridge, 2006; Burkhardt and Nerlich, 2010; Walker et al., 2021).

Empirical findings corroborate the notion that euphemisms shape audience judgments. In a large-scale experimental series (*N* = 3,081), Stanley and Neck (2025) found that euphemisms reduce the perceived severity of unethical actions such as bribery or fraud, leading to lower punitive responses. Notably, even when participants were later presented with accurate descriptions of the wrongdoing, the influence of euphemisms persisted. These results converge with findings by Walker et al. (2021), who demonstrated that widely used euphemisms can increase the social acceptability of unethical practices.

Research on linguistic framing consistently shows that susceptibility to framing effects varies across individuals. One such individual trait particularly relevant to framing susceptibility is Need for Cognition (NFC)—a stable dispositional trait capturing the extent to which individuals habitually engage in and enjoy effortful, elaborative thinking (Cacioppo and Petty, 1982). Individuals higher in NFC tend to process message content more deeply and systematically, showing greater sensitivity to argument quality and reduced reliance on peripheral or surface-level cues when forming evaluative judgments (Cacioppo et al., 1983). Consistent with this, Smith and Levin (1996) demonstrated that framing effects on decision making were observed only for individuals low in NFC, while those high in NFC remained unaffected by problem framing—showing that elaborative thinking reduces susceptibility to surface-level framing cues. LeBoeuf and Shafir (2003) further showed that high-NFC individuals, while not immune to initial framing, were more consistent across different frames of the same problem, reflecting greater adherence to normative principles when these are detectable. Together, these findings suggest that NFC moderates the degree to which linguistic framing shapes evaluative judgments. In the context of euphemistic framing, this suggests that individuals high in NFC may be more likely to engage with the underlying normative meaning of a described action rather than its linguistic packaging, and therefore show reduced susceptibility to the softening effect of euphemistic substitution.

Thus, euphemistic framing has persisting effects on moral evaluation at the behavioral level, but its underlying neural dynamics remain poorly understood. The present study addresses this question by comparing ERPs elicited by euphemized and non-euphemized vignettes of the same moral norm violations. This approach provides millisecond-level sensitivity to the neural processes engaged during the comprehension of norm violations and offers initial evidence on how euphemistic framing may shape their processing.

In the current study, we recorded electroencephalograms (EEG) and event-related potentials (ERPs) to investigate the role of euphemisms in the cognitive processing of norm violations. We used a set of vignettes describing a wide range of norm-violating actions, including lying, unethical behavior, and professional and social norm violations. The stimulus set was validated through a dedicated pretesting study (*n* = 50) confirming that the vignettes were perceived as at least weakly inappropriate in both euphemized and non-euphemized versions, and comprised a balanced mix of moral and conventional norm violations. Brain responses were probed while participants read vignettes systematically varied using euphemized, non-euphemized, and control critical words.

From an ERP perspective, two components are particularly relevant for capturing how readers process such norm-violating events in context: the N400 and the P600.

The N400 component, a negative deflection peaking around 400 ms, is associated with semantic incongruity. For instance, the word “nails” in “I like my coffee with nails” elicits a larger N400 because it conflicts with semantic expectations generated by the prior context (Kutas and Hillyard, 1980). Reduced N400 amplitudes occur when words are congruent with prior context, reflecting semantic memory organization (Federmeier and Kutas, 1999). Later work has shown that the N400 does not merely index semantic violations, but instead reflects a broader process of integrating word meaning with contextual and basic world knowledge (Hagoort et al., 2004; Nieuwland and Van Berkum, 2006).

Importantly, norm-violating behaviors, such as “Amanda is in the art museum, she is dancing,” elicit N400 responses, with cross-cultural variations in amplitude and topography observed between Asian and European participants (Mu et al., 2015). However, Leuthold et al. (2015) found that while knowledge-inconsistent words elicited a larger N400, moral norm violations evoked a distinct late positivity around 500 ms, suggesting different neural processes for semantic vs. moral violations.

P600 is another prominent positive component that occurs between 500 and 1,000 ms after semantically or syntactically incongruent critical words (Osterhout and Holcomb, 1992). Importantly, a number of studies reported the P600, instead of the N400, in response to some types of semantic violations including role-reversal and animacy violations (Hoeks et al., 2004; Kim and Osterhout, 2004; Kuperberg, 2007, Kuperberg and Jaeger, 2016; Nieuwland and Van Berkum, 2005; Van Herten et al., 2005; Van Petten and Luka, 2012). Semantically irregular but syntactically well-formed sentences may elicit the P600 but not N400 component. Researchers have suggested that the absence of the N400 may indicate that the semantic analysis leads to the illusion that the sentence is correct; however, with some delay, the reader realizes that the interpretation is invalid, as indicated by the stronger P600 amplitude. The role of the P600 in conflict resolution is evident in the syntactic phenomenon called “garden paths,” in which the final words of a sentence create an unexpected meaning, requiring the reader to revisit and reinterpret the beginning of the sentence (Gouvea et al., 2010). The latest “retrieval–integration neurocomputational model” suggests that the N400 reflects the retrieval of word-related semantic information, while the P600 component signals “the integration of this meaning into the unfolding utterance interpretation” (Seyednozadi et al., 2021).

Direct ERP evidence on euphemism comprehension remains scarce; therefore, we draw on adjacent ERP work on non-literal and other pragmatic phenomena. Because euphemisms are often more abstract and less norm-diagnostic than direct terms, ERP studies of indirectness and evaluative modulation—such as irony, metaphor, and metonymy—may offer a relevant process-level comparison. Such phenomena commonly elicit late positivities (P600/LPC) linked to pragmatic reanalysis and integration (Bambini et al., 2016; Regel et al., 2014), while N400 effects vary with predictability and contextual support (Van Petten and Luka, 2012).

However, different types of non-literal language engage distinct processing mechanisms, which limits the degree to which their ERP correlates directly transfer to euphemism comprehension. A comprehensive review of ERP research across figurative language types demonstrates that while earlier processing stages vary considerably, irony shows a robust neural correlate at later stages, typically reflected in late positive components associated with pragmatic reanalysis (Lai et al., 2023). For metaphor specifically, a scoping review of 62 experiments found that although a more negative N400 relative to literal counterparts is a consistent finding in the 200–600 ms window, results diverge substantially in the 500–1,000 ms window, with late component polarity varying as a function of stimulus emotionality and task demands (Baiocco et al., 2026). Euphemisms differ mechanistically from both: rather than creating cross-domain conceptual mappings or inverting propositional meaning, they substitute a direct expression with a softer alternative within the same semantic field, preserving the referent while reducing its normative diagnosticity. At the same time, like other forms of non-literal language, euphemisms require pragmatic reanalysis that goes beyond local lexico-semantic processing (cf. Lai et al., 2023), suggesting that late ERP components associated with integrative and evaluative reprocessing may be relevant across these phenomena.

Overall, previous studies have associated the N400 with predictability and plausibility of words in given contexts, while the P600 has been associated with conflict resolutions when an update of previous interpretation is needed (for a review, see Bornkessel-Schlesewsky and Schlesewsky, 2008; Kim and Osterhout, 2004; Van De Meerendonk et al., 2009; Kuperberg, 2007, Kuperberg and Jaeger, 2016; Van Herten et al., 2005; Van Schijndel and Linzen, 2021). Therefore, in the current study, we focused on the N400 and P600 components as potential signatures of the effects of euphemisms on the processing of norm violations.

In the current study, we recorded ERPs and collected behavioral severity ratings while participants read vignettes describing norm-violating events or neutral control events. Within the norm-violation conditions, the critical words describing the target action were manipulated linguistically, presenting either non-euphemized terms (e.g., ‘to lie’) or euphemized alternatives (e.g., ‘to conceal’). Control vignettes contained no norm violations. Using this design, we tested four primary hypotheses:

*H1.* At the behavioral level, we hypothesized that vignettes depicting norm violations with euphemized critical words would be perceived as less severe than those featuring non-euphemized critical words.

*H2.* At the neurophysiological level, we hypothesized that non-euphemized critical words would evoke stronger N400 and/or P600 amplitudes relative to euphemized critical words. An N400 modulation by euphemisms would suggest that euphemistic framing alters early lexico-semantic access or local plausibility during comprehension of norm-violating content. Conversely, a P600 modulation would indicate an effect on downstream, updating—specifically a discourse-level reevaluation of the event’s normative meaning, rather than on initial detection alone.

*H3.* Alternatively, if non-euphemized critical words are processed primarily as emotionally negative lexical items, negative valence may facilitate lexico-semantic retrieval, thereby attenuating rather than amplifying N400 amplitudes (Lai et al., 2024). Under this account, a pre-established affective context may reduce the cognitive effort required to process emotionally consistent words, leading to more efficient contextual integration instead of increased processing difficulty.

*H4.* Additionally, we examined whether individual differences in the Need for Cognition (NFC) modulate the behavioral and ERP correlates of euphemism processing. Because individuals higher in NFC are more inclined to engage in effortful and elaborative processing of message content, they demonstrate greater sensitivity to information quality (e.g., argument strength), relative to peripheral or surface-level cues (Cacioppo et al., 1983). On this basis, we hypothesized that higher NFC would be associated with a muted euphemism effect—evidenced by minimized differences in both behavioral severity ratings and ERP amplitudes between the euphemized and non-euphemized conditions.

## 2 Methods

### 2.1 Participants

Thirty-eight participants (18 females, aged 18–40 years) were recruited via online advertisements. The sample size was determined based on the power analyses conducted using G*power software. The repeated measures ANOVA would be sensitive to medium effect, as it is determined in Liang et al. (2024) when the alpha is accounted for 0.05 and power is 80%. All participants reported that they were right-handed, healthy, had normal or corrected-to-normal vision, had no history of psychiatric diagnoses, had no neurological disorders, and were not taking any prescribed medication. All participants were new to the experimental paradigm and were unaware of the real goal of the study. Prior to commencing the experiment, the participants provided written informed consent. The participants attended the study in exchange for monetary compensation equal to ~20 USD. The study design was approved by a local ethics committee in accordance with the Declaration of Helsinki.

### 2.2 Materials and design

#### 2.2.1 Norm violation task

For the current study, we used a set of vignettes (scenarios) depicting norm violations. The set of 40 vignettes was modified using three types of critical words: (1) non-euphemized critical words signaling the norm violations (non-euphemized trials, *n* = 40), (2) euphemized critical words signaling the same norm violations (euphemized trials, *n* = 40), and (3) neutral critical words signaling no norm violations (control trials, *n* = 40). The English translation of all vignettes is available in Supplementary Table S2. For example, in the non-euphemized trials, the participants were exposed to the following vignette (English translation): “*The doctor asked the blood donor about his HIV status. The donor decided to talk about it.*” In the euphemized trials, only the critical word was altered: “*The doctor asked the blood donor about his HIV status. The donor decided to conceal it.*” Meanwhile, in the control trials with no norm violations, the socially appropriate (or neutral) critical word was used: “*The doctor asked the blood donor about his HIV status. The donor decided to talk about it.*” At the end of each trial, participants evaluated norm violations using a 6-point Likert scale.

The vignettes represented a wide spectrum of norm violations that could elicit moral judgments. Presented in a concise news headline-style format, the vignettes were crafted to provoke assessments while imitating brief and impactful media messages. When crafting the vignettes, we purposefully omitted any references to specific names, well-known public figures, or events. With this, we aimed to minimize the potential personal affective responses that may influence participants’ moral judgments. Overall, the study was designed to elicit third-party moral judgments; therefore, the vignettes contained no personal references to the participants. The EEG was recorded during the presentation of all randomized, non-euphemized, euphemized, and control trials. At the end of the study, the participants completed the NFC scale.

The euphemized stimuli were selected to be semantically related to the corresponding direct expressions and to preserve the same general event domain, while presenting the event in a less explicit and less evaluatively direct form (e.g., Supplementary Table S14). The classification of all stimulus pairs as euphemized/non-euphemized was based on empirical pretesting—we conducted a study in which a separate group of participants (*n* = 32, 24 females, aged 18–43 years) evaluated 20 unique word pairs consisting of non-euphemized and euphemized critical words. On average, participants reached consensus on 18 out of 20 word pairs, consistently selecting the same expression as the softer alternative. The validated word pairs were subsequently used to construct 40 vignettes describing a range of norm-violating scenarios.

To validate it, we conducted a dedicated pretesting study (*n* = 50) in which separate group of participants evaluated all stimuli used in the reported experiment. Each vignette was presented in full, and participants completed two tasks for each stimulus. First, they rated the perceived inappropriateness of the described behavior on the scale used in the main experiment (1 = appropriate, 2 = weakly inappropriate, 3 = strongly inappropriate). Second, they categorized each stimulus as a moral or conventional norm violation based on established criteria (Huebner et al., 2010), including authority-independence (e.g., “Would this still be wrong even if it were permitted by law?”). The study used a within-subjects design with all stimuli presented in both euphemized and non-euphemized versions in a randomized order. Inappropriateness ratings confirmed that stimuli in both experimental conditions were perceived as at least weakly inappropriate: mean ratings were 2.01 (SD = 0.47) for euphemized and 2.14 (SD = 0.43) for non-euphemized versions. Only three euphemized items fell below 1.5; all others exceeded this threshold. Categorization results showed a balanced stimulus set (33 moral and 47 conventional violations), though the distribution varied by condition: the non-euphemized set comprised 17 moral and 23 conventional violations, while the euphemized set comprised 16 moral and 24 conventional violations.

To evaluate whether the experimental conditions differed in lexical predictability, we conducted an exploratory language-model surprisal analysis for all 120 critical words. Surprisal was estimated using the Russian causal language model ai-forever/rugpt3medium_based_on_gpt2 (Zmitrovich et al., 2024). For each item, the conditional probability of the critical word was calculated given the preceding vignette context. Surprisal was defined as −log₂ *P*(word|preceding context), expressed in bits following recent psycholinguistic frameworks that utilize large language model outputs as computational estimates of contextual predictability and predictors of N400 amplitude (Michaelov et al., 2024). When a critical word was split into multiple subword tokens, word-level surprisal was computed as the sum of token-level surprisals. Crucially, the analysis did not reveal statistically significant differences across the non-euphemized (mean = 9.67 bits), euphemized (mean = 10.34 bits), and control conditions (mean = 10.75 bits), with all pairwise comparisons yielding *p* > 0.20 (see Supplementary Figure S1). Furthermore, the number of subword tokens also did not vary significantly between conditions, confirming that the surprisal metrics were not confounded by systematic discrepancies in tokenization.

#### 2.2.2 Experimental procedure

Each participant was exposed to 120 vignettes. Following the presentation of each vignette, the participants were prompted to rate the level of their appropriateness using a 6-point Likert scale, ranging from 0 (no violation of norms) to 5 (severe violation), by moving the cursor with a mouse (Figure 1). There was no time constraint that allowed the participants to proceed to the next stimulus at their own pace. The participants were instructed to attentively follow the on-screen information, focus their gaze at the center of the screen, and answer the question at the end of the trial. The vignettes were presented word by word (stimulus duration = 500 ms, randomized interstimulus interval = 333–660 ms); during the interstimulus interval, the fixation cross was presented on the screen. The vignettes were presented in pseudo-random order (intertrial interval = 333–660 ms), ensuring the minimum time difference between the same vignette type presentation of 120 s.

**Figure 1:**
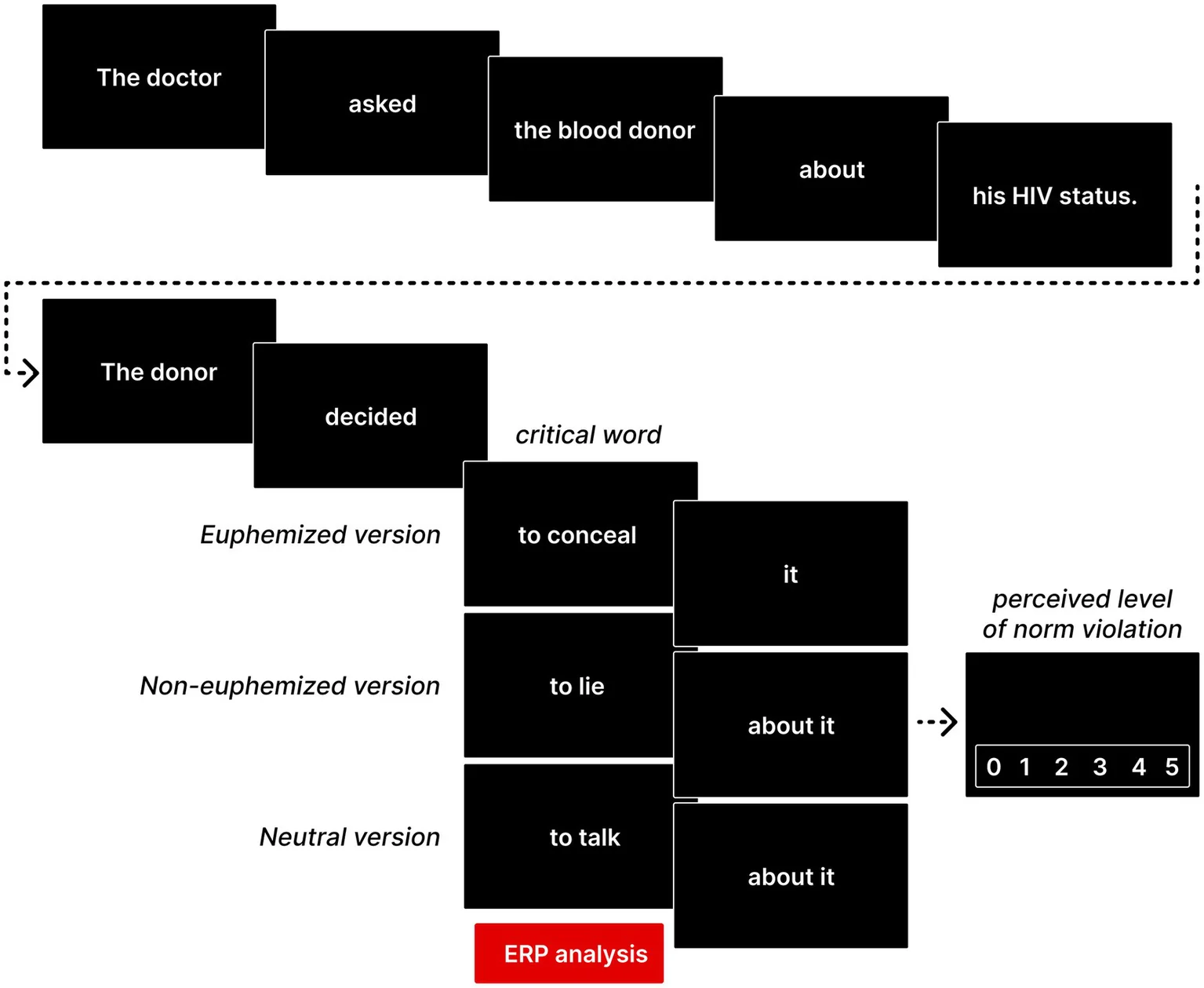
Schematic representation of the experimental trial sequence. Each trial begins with the contextual vignette framing the social scenario, followed by the critical word presentation screen. The target action is isolated on screen and linguistically manipulated into either a non-euphemized term (e.g., ‘to lie’), a euphemized alternative (e.g., ‘to conceal’), or a neutral control expression. Following vignette completion, participants evaluate the severity of the action using a forced-choice 6-point Likert scale, ranging from 0 (*Not severe at all*) to 5 (*Extremely severe*).

#### 2.2.3 EEG recording

EEG was recorded using a 64-channel EEG setup (BRAINAMP DC) with electrodes positioned on the scalp according to the 10–20 system (Klem et al., 1999). Impedances were kept <10 kΩ. Eye movements were recorded with four electrodes placed at both the lateral canthi and below the left eye. The EEG signals were referenced to the mastoids. To ensure accuracy, four electrodes were specifically allocated for ocular correction, and the data were sampled at a rate of 500 Hz.

All preprocessing and processing steps were performed in MNE Python (Gramfort et al., 2013; see also Luck, 2005). The EEG was bandpass offline filtered in the 1–45 Hz frequency range. The EEG data were also notch-filtered (band-stop filter: 49–51 Hz). To verify that the observed effects were not driven by filter settings, we repeated the analysis with a 0.1–30 Hz bandpass filter; the pattern of results was unchanged (see Supplementary Figures S2, S3). The data were inspected for artifacts, and amplitudes exceeding ±100 μV were excluded from further analysis and then interpolated based on neighboring channels. Overall, 7% of the epochs were removed from the analysis. To further remove physiological artifacts, we used independent component analysis (ICA) decomposition using the fast ICA method. The number of ICA components used for decomposition was set to explain 99% of the variance. Components containing eye-related artifacts were deleted from the analysis.

The data were epoched from −250 ms to 1,000 ms after the onset of critical words. The set of epochs was defined for each of the event types (control, with non-euphemized and euphemized trials; see “Materials and Design”), and the signal was baseline corrected based on the mean activity within a −250 to 0 ms time window.

To study the N400 and P600 components, we analyzed ERPs within 300–500 ms and 500–700 ms, respectively. This study was not pre-registered.

### 2.3 Statistical analysis

#### 2.3.1 Behavioral data analysis

We conducted the Levene test to assess homogeneity and employed the Shapiro–Wilk normality test to evaluate the variance’s normality for the behavioral data. To study the effect of euphemisms on perceived severity of norm violations, we utilized the Kruskal–Wallis test with the factor of condition (non-euphemized, euphemized, or control critical words).

#### 2.3.2 Lexical covariates analysis

To assess whether psycholinguistic properties of the critical words could account for the observed behavioral and ERP effects, we fitted linear mixed-effects models with experimental condition as the predictor of interest and word-level covariates—word frequency, valence, word length, and arousal—entered to statistically control for their contribution. An additional model included socialness as a covariate in place of arousal; as socialness ratings were not available for all stimulus words, these two variables were entered in separate models rather than simultaneously (*N* = 4,440 and *N* = 3,663 respectively). Separate models were fitted for behavioral severity ratings and P600 amplitude as dependent variables. The euphemized condition served as the reference category in all models.

#### 2.3.3 ERP analysis

For the statistical analysis, ERPs were time-locked to the onset of the critical word and computed separately for the non-euphemized, euphemized, and control conditions. Statistical analyses focused on two predefined time windows: 300–500 ms for the N400 and 500–700 ms for the late positivity (P600) range. To evaluate the global neural response, we analyzed the ERPs averaged across all 60 scalp electrodes; EOG channels were excluded from this averaging, and no other recording sites were omitted. The mean number of trials retained per participant after preprocessing was highly comparable across conditions: 36.84 for non-euphemized, 37.16 for euphemized, and 36.95 for control trials. To track condition effects over time, we first applied a data-driven temporal cluster-based permutation *F*-test to the ERPs averaged across all 60 scalp electrodes. The permutation distribution was constructed using 1,024 iterations with an initial cluster-forming threshold of *F* = 1.70 (based on temporally adjacent samples). Planned dependent-samples t-tests were subsequently conducted for the predefined pairwise contrasts within the N400 and late positivity/P600 epochs: non-euphemized versus euphemized, non-euphemized versus control, and euphemized versus control. For each participant and condition, mean amplitudes were extracted within each predefined latency window; peak amplitude was additionally extracted within the 470–750 ms window as a supplementary index. Statistical outliers were identified using Tukey’s fence method (Tukey, 1977), applied separately to each pairwise contrast. For the non-euphemized versus euphemized contrast, 34 participants were retained for the mean-amplitude analysis and 32 for the peak-amplitude analysis; for the non-euphemized versus control contrast, 36 and 37 participants, respectively; and for the euphemized versus control contrast, 35 and 36 participants, respectively. Normality was verified using Shapiro–Wilk tests prior to parametric evaluation.

Finally, Pearson correlation coefficients were used to explore potential associations between Need for Cognition (NFC) scores and both behavioral and ERP metrics. For the ERP analyses, we examined correlations between NFC scores and the magnitude of the late positivity/P600 effect, operationalized as the amplitude difference between the non-euphemized and euphemized conditions. In alignment with the primary ERP pipeline, these correlational analyses were performed on amplitudes collapsed across all 60 scalp electrodes.

## 3 Results

### 3.1 Behavioral results

The results of the Shapiro–Wilk normality indicated that the ratings of norm violations followed a normal distribution in the euphemized trials (*p* = 0.11), while the ratings in the non-euphemized (*p* = 0.029) and control (*p* = 0.01) trials were not normally distributed. Levene’s test indicated that the homogeneity assumption was not violated: *F*(2, 111) = 0.24, *p* = 0.79. The Kruskal–Wallis test confirmed our behavioral hypothesis H1 and suggested differences in the participants’ evaluation of the norm violations depicted in vignettes containing non-euphemized, euphemized, and neutral critical words: *H* = 81.72, *p* < 0.001, *ε*^2^ = 0.72. The participants perceived the highest level of norm violation in the non-euphemized trials (M = 4.09, SD = 0.49) compared to the euphemized (M = 3.61, SD = 0.56) and control trials (M = 0.74, SD = 0.53). *Post hoc* comparisons using the Conover–Iman test with Holm correction confirmed a significant difference in the perceived level of norm violations between vignettes with euphemized critical words versus control critical words (*p* < 0.001). Similarly, perceived severity of norm violation significantly differed between vignettes with non-euphemized critical words and control critical words (*p* < 0.001), and between vignettes with non-euphemized and euphemized critical words (*p* < 0.001) (Figure 2). Overall, the behavioral results confirmed that euphemisms significantly reduced perceived severity of norm violations compared to non-euphemized and neutral words.

**Figure 2:**
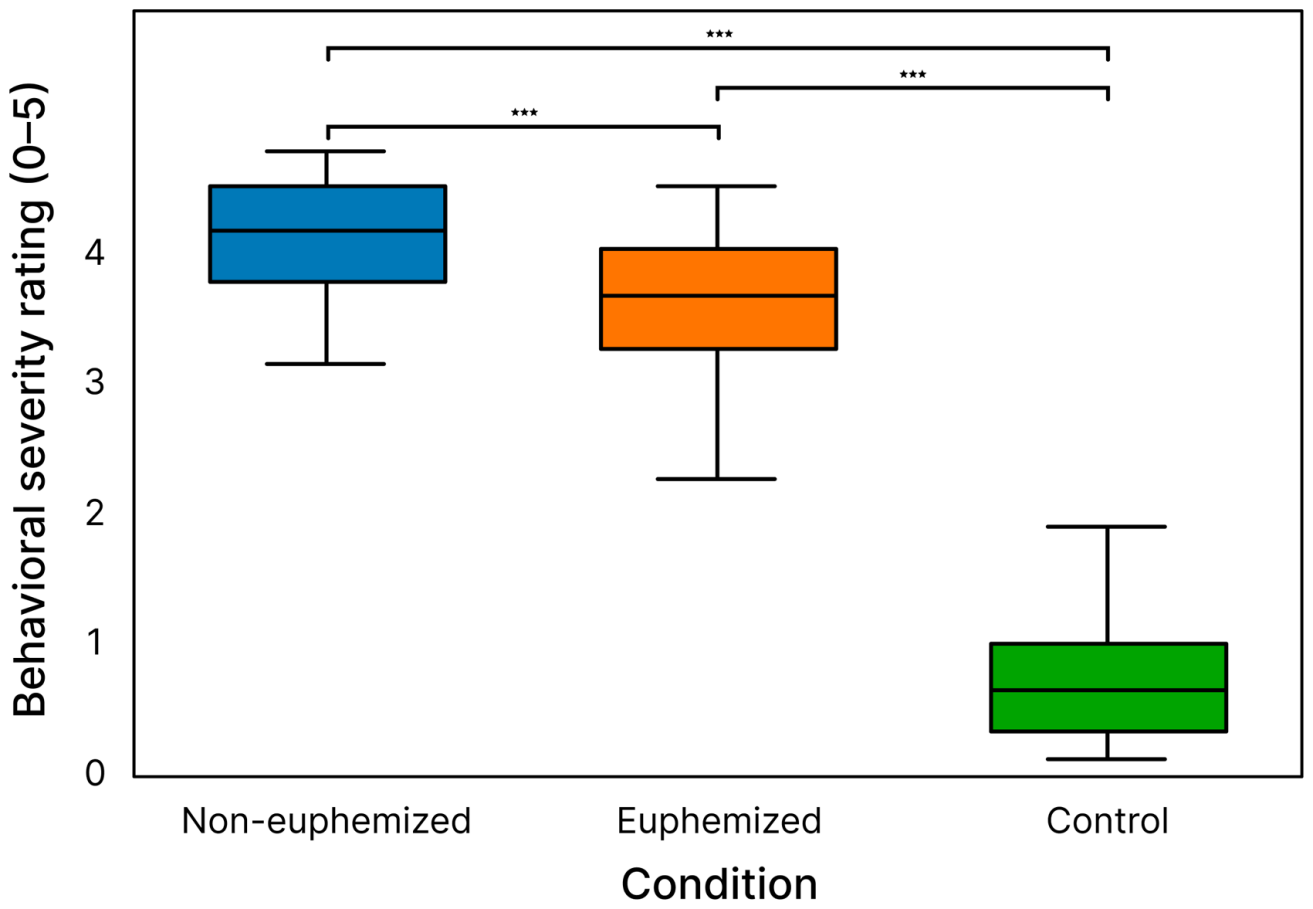
Behavioral severity ratings across experimental conditions. Boxplots illustrate the distribution of severity ratings for the non-euphemized, euphemized, and control vignettes. Horizontal lines indicate the median, boxes represent the interquartile range (IQR), and whiskers denote 1.5 × IQR. ***Asterisks indicate statistically significant pairwise differences (*p* < 0.001).

### 3.2 Results of lexical covariates analysis

To examine potential psycholinguistic confounds, we first compared word length and word frequency across all three conditions. Descriptive statistics are provided in Supplementary Tables S8, S9. Non-euphemized words had a mean length of 8.05 letters (SD = 2.10) and a mean frequency of 18.28 (SD = 18.56); euphemized words had a mean length of 9.98 letters (SD = 2.85) and a mean frequency of 18.05 (SD = 21.52); control words had a mean length of 8.98 letters (SD = 2.49) and a mean frequency of 101.59 (SD = 196.19). Pairwise *t*-tests comparing the non-euphemized and euphemized conditions revealed a significant difference in word length (*t* = −3.44, *p* < 0.001) but no significant difference in word frequency (*t* = 0.05, *p* = 0.96).

To assess whether these lexical properties contributed to behavioral severity ratings, we fitted linear mixed-effects models with Likert ratings as the dependent variable, experimental condition as the predictor of interest, and word frequency, valence, word length, and arousal entered as covariates. The euphemized condition served as the reference category in all models. All four covariates reached significance: word frequency (*β* = −0.000, SE = 0.000, *p* = 0.003), valence (*β* = −0.246, SE = 0.042, *p* < 0.001), word length (*β* = 0.016, SE = 0.007, *p* = 0.036), and arousal (*β* = 0.103, SE = 0.044, *p* = 0.020), though their coefficients were small in magnitude. Critically, the effect of experimental condition remained highly significant after controlling for all covariates (*β* = 0.425, SE = 0.048, *p* < 0.001; see Supplementary Table S4). When socialness was included as a covariate in place of arousal, it did not reach significance (*β* = −0.024, SE = 0.017, *p* = 0.152), and the condition effect persisted (*β* = 0.374, SE = 0.052, *p* < 0.001; see Supplementary Table S5). As socialness ratings were not available for all stimulus words, arousal and socialness were entered in separate models rather than simultaneously (*N* = 4,440 and *N* = 3,663 respectively).

To assess whether lexical and affective properties contributed to P600 amplitude, we fitted equivalent linear mixed-effects models with P600 amplitude (μV) as the dependent variable, with all three conditions included as levels of the condition factor (euphemized as reference category), and word frequency, word length, valence, and arousal entered as covariates. Of these, only word length reached significance (*β* = −0.236, *p* = 0.001), while word frequency (*β* = 0.001, *p* = 0.441), valence (*β* = −0.238, *p* = 0.433), and arousal (*β* = 0.017, *p* = 0.956) did not. Additionally, socialness did not significantly predict P600 amplitude (*β* = 0.192, *p* = 0.127), and no significant difference in socialness was found between experimental conditions [*t*(58.83) = 0.9445, *p* = 0.349]. Critically, the effect of experimental condition remained significant after controlling for all covariates—in both the arousal model (*β* = 1.007, *p* = 0.004; see Supplementary Table S6) and the socialness model (*β* = 1.006, *p* = 0.009; see Supplementary Table S7). As socialness ratings were not available for all stimulus words, arousal and socialness were entered in separate models rather than simultaneously (*N* = 4,440 and *N* = 3,663 respectively).

### 3.3 ERP results

ERPs to critical words differed primarily in the late positive time range (Figure 3). In the grand-average waveforms, a small transient divergence between conditions was visible around 300–400 ms, whereas the largest separation occurred between approximately 400 and 700 ms, with non-euphemized critical words eliciting larger positive amplitudes than euphemized and control critical words.

**Figure 3:**
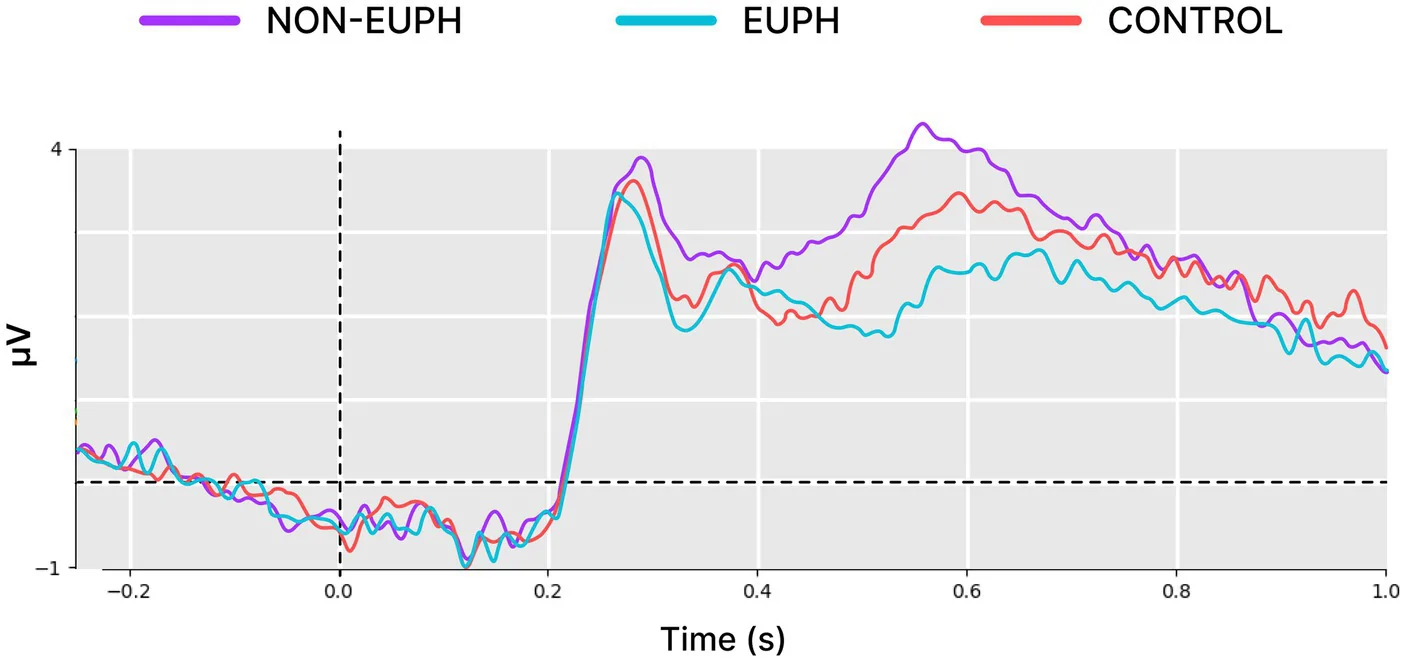
Grand-average ERP waveforms for critical words across experimental conditions. Waveforms are time-locked to critical word onset, indicated by the vertical dashed line at 0 ms, and averaged across the 60 scalp electrodes included in the ERP analysis. The non-euphemized, euphemized, and control conditions are represented by distinct line profiles. Amplitudes are displayed in microvolts (μV), with positive values plotted upward.

The cluster-based permutation *F*-test revealed significant temporal clusters for all three pairwise condition contrasts in the late time window (Figure 4). For the non-euphemized versus euphemized contrast, a significant cluster emerged between 442 and 666 ms (*p* = 0.007). Significant clusters were also manifested for the non-euphemized versus control contrast between 404 and 590 ms (*p* = 0.019), and for the euphemized versus control contrast between 496 and 656 ms (*p* = 0.027). No significant condition differences were observed within the predefined latency N400 window. Overall, euphemized critical words elicited a significantly attenuated late P600-like positivity relative to both non-euphemized and neutral control words.

**Figure 4:**
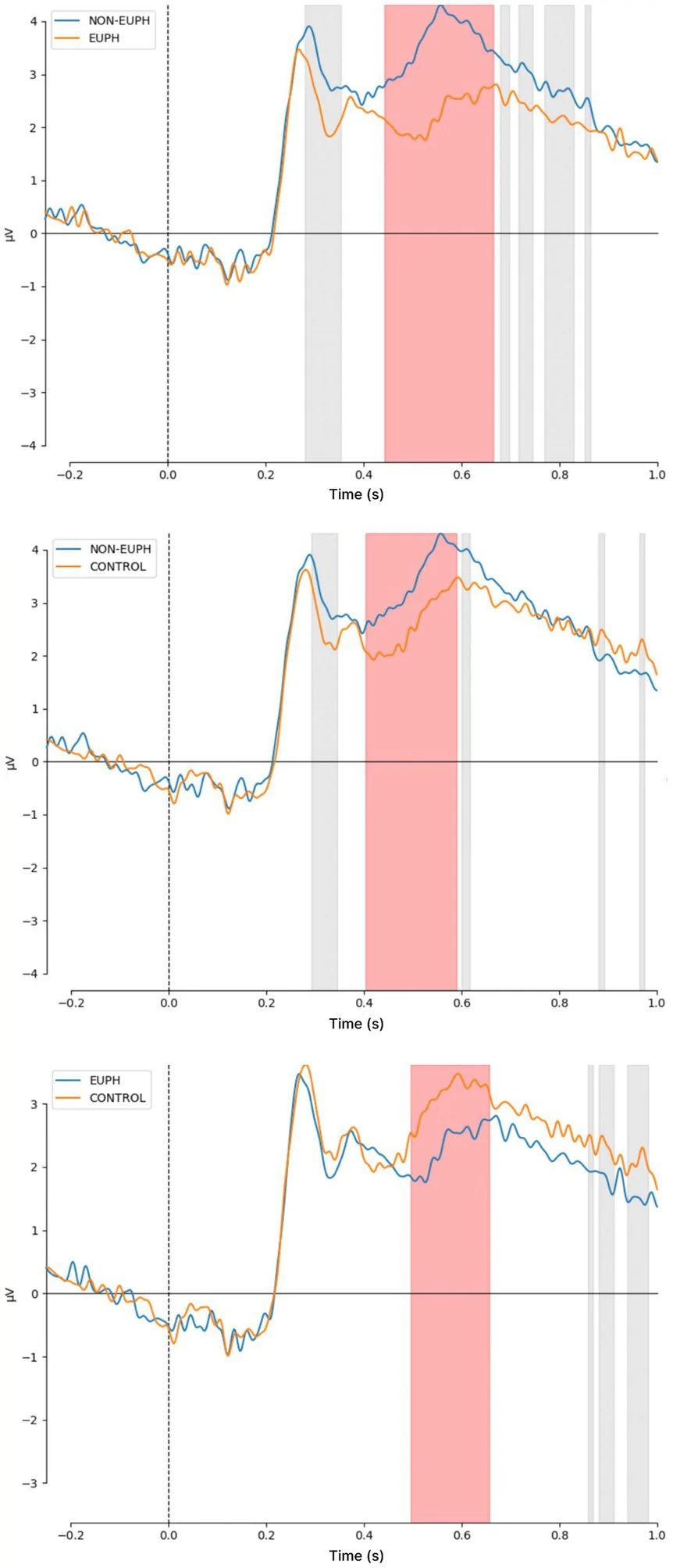
Temporal clusters identified by the cluster-based permutation analysis for pairwise ERP contrasts. Panels display grand-average ERP waveforms for the two conditions in each of the three pairwise contrasts: non-euphemized versus euphemized contrast (top), non-euphemized versus control contrast (middle), and euphemized versus neutral control critical words (bottom). Waveforms are time-locked to critical word onset, indicated by the vertical dashed line at 0 ms, with amplitudes plotted in microvolts (μV). Red shaded intervals indicate statistically significant temporal clusters; gray shaded intervals indicate non-significant clusters. Statistically significant clusters emerged for the non-euphemized versus euphemized contrast (442–666 ms), the non-euphemized versus control contrast (404–590 ms), and the euphemized versus control contrast (496–656 ms).

Difference-wave and topographical plots further characterized the morphology of this effect (Figure 5). The largest positive deflection peaked at approximately 530–550 ms, and the corresponding topographical maps revealed a broadly distributed positivity across the scalp, with the most pronounced differences over frontocentral and frontoparietal regions. Planned dependent-samples *t*-tests in the late positivity/P600 window showed significant differences between all pairwise contrasts for mean amplitudes: non-euphemized versus euphemized, *t*(33) = 3.643, *p* < 0.001; non-euphemized versus control, *t*(35) = 2.537, *p* = 0.016; and euphemized versus control, *t*(34) = −2.875, *p* = 0.006 (Supplementary Table S1). Peak-amplitude analyses in the same window showed the same pattern of significant pairwise differences (all *p* < 0.05; see Supplementary Table S1). Thus, the statistically reliable (euphemism-related) ERP differences were concentrated in the late positivity/P600 time range rather than the N400 window.

**Figure 5:**
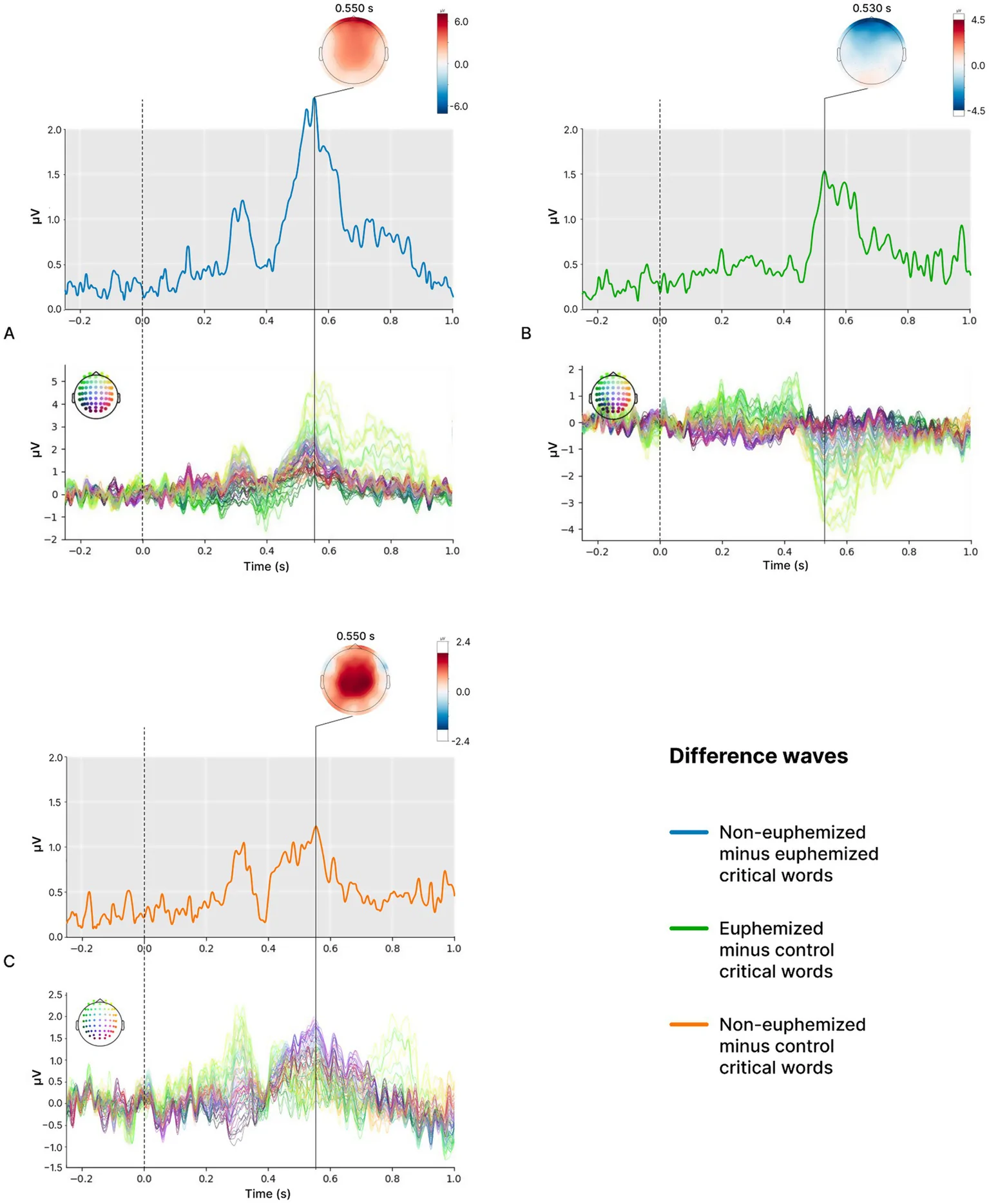
Difference waves and scalp topographies for pairwise ERP contrasts. Panels display three pairwise difference waves: **(A)** non-euphemized minus euphemized, **(B)** euphemized minus neutral control, and **(C)** non-euphemized minus neutral control critical words. For each contrast, the upper profile shows the difference wave averaged across the 60 scalp electrodes included in the ERP analysis, and the lower plot displays electrode-wise difference waveforms. Scalp topographies illustrate the spatial distribution of difference-wave amplitudes at the indicated peak latencies. Waveforms are time-locked to critical word onset, indicated by the vertical dashed line at 0 ms.

### 3.4 Personal traits

We used Pearson’s correlation test to investigate the relationship between behavioral/ERP correlates of euphemism processing and NFC scores. We found no significant correlation between individual NFC scores and the differential evaluation of norm violations (mean perceived norm violations in non-euphemized *minus* mean perceived norm violations in euphemized vignettes): *r* = 0.05, *p* = 0.364. We also found no significant correlation of the individual NFC scores with the peak/mean amplitudes of the differential P600 (ERPs to non-euphemized critical words *minus* ERPs to euphemized critical words): *r* = −0.2, *p* = 0.131; *r* = −0.1, *p* = 0.320 for the mean amplitudes and peak amplitudes, respectively.

## 4 Discussion

In the current study, we investigated the neural correlates underlying the processing of euphemized vignettes depicting norm violations. We compared event-related potentials (ERPs) to vignettes modified with euphemized, non-euphemized, or neutral critical words. Behaviorally, participants rated norm violations framed with euphemisms as significantly less severe than those described with non-euphemized language, providing full support for hypothesis H1. At the electrophysiological level, the results offered partial support for hypothesis H2: non-euphemized critical words elicited a significantly larger P600 component compared to both euphemized and control conditions, while no reliable euphemistic framing-related modulation of the N400 was observed. The temporal and topographical morphology of the ERP response constrains the interpretation of the observed effect. Across the grand-average waveforms, difference waves, and topographical maps, the most consistent euphemism-related modulation manifested within the late P600 latency window rather than the early N400 range. Difference-wave plots revealed a transient, statistically non-significant divergence around 300 ms, whereas the dominant frontocentral effect emerged downstream within the P600 time range as a positive deflection peaking between approximately 530 and 550 ms. Together with the absence of a reliable N400 modulation, this pattern supports a cautious characterization of the observed effect as a late positivity in the P600 range rather than an early lexico-semantic mismatch response. This interpretation aligns with recent evidence demonstrating that pragmatic incongruity can elicit a P600 in the absence of an N400. For example, Blache et al. (2026) showed that incongruent conversational feedback did not elicit an N400 response but instead produced a P600 effect, which they attributed to pragmatic-level processing. In the present study, euphemistic framing may similarly have modulated the downstream integration of the event’s normative meaning within the discourse context, rather than generating an early lexico-semantic expectancy effect.

The N400 component was originally described as a marker of local semantic incongruity, elicited by anomalous words violating sentence-level expectations (Kutas and Hillyard, 1980). Later work expanded this perspective, demonstrating that N400 amplitudes reflect the ease of accessing word-associated information from long-term memory, modulated by contextual pre-activation (Brouwer et al., 2016; Kutas and Federmeier, 2011). Amplitudes are typically larger for semantically unexpected but plausible words (Federmeier and Kutas, 1999), world-knowledge violations (Hagoort et al., 2004), and certain social norm violations (Mu et al., 2015; Luo et al., 2019).

In classic norm violation paradigms (e.g., Mu et al., 2015), the critical word creates an immediate conflict at the level of lexical associations—"museum” pre-activates expectations incompatible with “dancing,” producing a rapid associative mismatch indexed by the N400. When words remain locally compatible and normative conflict arises primarily during broader discourse or social interpretation, early retrieval-related effects can be attenuated or absent (Klingvall and Heinat, 2024). In our paradigm, both euphemized and non-euphemized critical words are semantically congruent continuations of the vignette at the lexical level. The normative conflict emerges only during integration of the broader moral meaning of the described action—a later higher-order process more consistent with the P600 than with early lexico-semantic retrieval indexed by the N400.

This pattern aligns with accounts positing that the N400 is most sensitive to facilitated lexico-semantic processing under fulfilled predictions, whereas P600 reflects higher-level updating when strong predictions are violated or revised (Kuperberg et al., 2020). We note that robust N400 effects for norm violations have been consistently reported across multiple validated paradigms (Mu et al., 2015; Goto et al., 2022; Salvador et al., 2020). Therefore, the absence of N400 modulation in the present study should not be interpreted as a challenge to the general literature on norm violation processing; rather, it suggests that N400 amplitude may not be sensitive to the specific contrast between euphemized and non-euphemized framing of identical violations.

The P600 was initially linked to syntactic reanalysis and repair in response to violations of phrase structure, agreement, tense, or argument structure (Osterhout and Holcomb, 1992; Hagoort et al., 1993; Ainsworth-Darnell et al., 1998; Allen et al., 2003). Subsequent research, however, revealed P600-like effects in semantically and pragmatically anomalous but syntactically well-formed sentences, including role reversals, semantic conflicts, and cases requiring reevaluation after temporary misinterpretations (Kim and Osterhout, 2004; Kuperberg et al., 2003; Kuperberg, 2007; Stroud and Phillips, 2012; Kaan et al., 2000).

Contemporary views therefore frame the P600 as a broad index of integration effort and discourse-level updating, particularly when conflicts are potentially resolvable through reinterpretation (Aurnhammer et al., 2021, 2024; Delogu et al., 2019; Klingvall and Heinat, 2024). Late positivity can also emerge when evaluation depends on integrating broader context to reach a good/bad judgment, rather than on a basic world knowledge (Leuthold et al., 2015). These findings support the idea that P600 reflects integrative effort when local coherence is preserved, but broader interpretation is required.

In line with this framework, euphemized and non-euphemized vignettes in our study were lexico-semantically compatible at the word level but differed markedly in normative diagnosticity. The normative implications of the described actions likely became salient primarily during later-stage integration into a broader social and moral context. The larger P600 for non-euphemized critical words is thus consistent with increased demands for discourse-level updating and evaluative reappraisal when an action is explicitly labeled as norm-violating. By contrast, euphemized critical words reduce the immediate normative salience of the act, allowing for less condemnatory or more ambiguous construals and thereby attenuating the magnitude of late integrative updating. In this way, euphemistic framing appears to function as a “cognitive buffer,” dampening the neural effort associated with integrating norm-violating content at the stage of evaluative reinterpretation.

This pattern is consistent with Van Berkum et al. (2009), who demonstrated that morally objectionable words embedded in sentences elicit a late positive component, suggesting that moral evaluation engages neural processes distinct from standard lexico-semantic integration. In line with this, our results suggest that normative evaluation in context is primarily indexed by later integrative processes rather than early semantic retrieval. Crucially, euphemistic framing appears to modulate the magnitude of this late positivity rather than its presence.

An alternative interpretation is that the observed late positivity reflects emotional arousal rather than integrative reappraisal—characterizing the effect as a Late Positive Potential (LPP) rather than a P600. To test this directly, we conducted linear mixed-effects modeling on P600 amplitude including arousal and valence scores of the critical words as covariates. Neither arousal (*β* = 0.017, *p* = 0.956) nor valence (*β* = −0.238, *p* = 0.433) significantly predicted P600 amplitude. Critically, the condition effect remained significant and virtually unchanged after controlling for these factors (*β* = 1.007, *p* = 0.004). These results argue against a purely emotional arousal account and support our interpretation of the late positivity as reflecting discourse-level integrative reappraisal of norm-violating content.

We also evaluated whether this neurophysiological euphemism-related effect could be attributed to systematic differences in lexical predictability. Our exploratory language-model surprisal analysis revealed no reliable differences across conditions, confirming that the late positivity was unlikely to be driven by word-level expectancy variations. By ruling out basic lexical-semantic confounds, these computational metrics lend further support to a downstream discourse-pragmatic or evaluative integration account of the observed P600 modulation.

Interestingly, our control critical words, which signal no norm violations, elicited a larger P600 than euphemized critical words. Since the majority of the trials (in two experimental conditions) signaled norm violations, the control stimuli may stand out and require a relatively more complex integration into the overall context of the norm violation tasks.

We found no significant correlation between participants’ inclination toward effortful cognitive activities (NFC scores) and both—norm violation evaluations and ERP amplitudes differences (H4). This may suggest that in our tasks, both initial reactions and evaluations were primarily rapid, occurring within online language processing without the deliberate analytical effort typically associated with NFC, whereas controlled reasoning played a secondary role in the *post hoc* justification of these judgments (for the same reasoning, see Conway and Gawronski, 2013; Greene et al., 2001). Future studies should explore experimental designs that promote deeper analytical processing to better understand the role of NFC in resistance to linguistic manipulation in moral judgments.

### 4.1 Limitations

The present stimulus set contains a mix of moral and conventional norm violations, as confirmed by our pretesting study. Whether the effects of euphemistic framing on P600 amplitude vary as a function of violation type remains an open question that future research should address systematically.

The term “euphemism” as used in this study reflects an operational definition grounded in empirical pretesting rather than a single theoretical account, as definitions of euphemism vary across the linguistic literature. Importantly, our stimuli were originally developed in the language native to participants and translated into English for this manuscript. While we prioritized accuracy, some language-specific connotations or pragmatic nuances may have been subtly altered during the translation process. Cross-linguistic replication using language-specific stimulus sets would help establish the generalizability of the present findings.

A further limitation is that the semantic distance between the non-euphemized and euphemized critical words was not independently quantified. Future studies should assess this dimension explicitly—perhaps via behavioral semantic-differential scales or computational vector-space modeling—to examine whether the degree of euphemistic attenuation modulates neural responses as an item-level covariate or an additional experimental factor.

While the present study controlled for word frequency, length, valence, arousal, and socialness, future work may benefit from additionally examining psycholinguistic dimensions such as morphological complexity, concreteness, and cloze probability, particularly as standardized norms become available for Russian-language stimuli. Although our exploratory language-model surprisal analysis did not reveal statistically significant differences in model-based lexical predictability between the experimental conditions, such estimates cannot fully substitute for item-level cloze-probability norming or contextual-fit ratings.

## 5 Conclusion

Research on the neural correlates of morally objectionable language has demonstrated that moral evaluation engages distinct neural processes from standard lexico-semantic integration, typically reflected in late positive components rather than the N400 (Van Berkum et al., 2009). The present study extends this line of work by showing that euphemistic framing modulates the magnitude of this late positivity—specifically by attenuating the P600 elicited by norm-violating content without eliminating it. Throughout human history, euphemisms have framed narratives to subtly influence our judgments (Allan and Burridge, 1991). In the current study, participants rated norm violations in vignettes with euphemized critical words as significantly less severe than those with non-euphemized critical words. In parallel, non-euphemized critical words evoked a significantly larger P600 component than euphemized critical words, while no comparable effect of euphemistic framing emerged on the N400. These ERP results suggest that euphemisms primarily modulate late-stage structural integration, reanalysis, and evaluative reappraisal of perceived norm violations (indexed by the P600), rather than early detection of normative conflict (N400). The attenuated P600 for euphemized descriptions may also be regarded as reduced cognitive effort required to reconcile the critical word with the unfolding moral context.

In the modern media environment, framing can systematically shape how events and actions are perceived. Identifying the neurocognitive mechanisms by which euphemisms shift normative judgments is an important step toward the development of countermeasures against their manipulative use. The present study provides initial evidence in this direction.

## Data Availability

The datasets presented in this study can be found in online repositories. The names of the repository/repositories and accession number(s) can be found at: https://osf.io/xbks2/overview.
