# Supplementary material for: Euphemisms modulate the neural processing of norm violations
Source: Front Hum Neurosci. 2026 Sep 23;20:1917736. doi: 10.3389/fnhum.2026.1917736 (PMC13642805; doi:10.3389/fnhum.2026.1917736)
Supplement: Supplementary file 1 [file Data_Sheet_1.docx]

# ***Supplementary Materials***

Supporting information for:

Euphemisms Modulate the Neural Processing of Norm Violations

Yulia Gorodnicheva^*^, Eliana Monahhova, Qiang Shen, Amir Bekim, Vasily Klucharev

*Yulia Gorodnicheva

E-mail: gorodni4evayulia@gmail.com

**Supplementary Materials	1**

**Supplementary Table S1. The table of pairwise t-test for P600 to non-euphemized, euphemized and control critical words	2**

**Supplementary Table S3. Covariates Dictionary	6**

**Supplementary Table S4. Results for Linear Mixed-Effects Model with the LIKERT ratings as the dependent variable (“emot” – Non-euphemized, “less” – Euphemized and “filler” – Neutral).	7**

**Supplementary Table S5. Results for Linear Mixed-Effects Model with the LIKERT ratings as the dependent variable (“emot” – Non-euphemized, “less” – Euphemized and “filler” – Neutral).	7**

**Supplementary Table S6. Results for Linear Mixed-Effects Model with the P600 amplitude (μV) as the dependent variable (“emot” – Non-euphemized, “less” – Euphemized and “filler” – Neutral).	8**

**Supplementary Table S7. Results for Linear Mixed-Effects Model with the P600 amplitude (μV) as the dependent variable (“emot” – Non-euphemized, “less” – Euphemized and “filler” – Neutral).	8**

**Supplementary Table S8. Descriptive statistics for word length.	9**

**Supplementary Table S9. Descriptive statistics for word frequency in literature.	9**

**Supplementary Table S10. Categorization results for non-euphemized vignettes: moral vs. conventional norm violation classification based on pretesting (n = 50).	9**

**Supplementary Table S11. Categorization results for euphemized vignettes: moral vs. conventional norm violation classification based on pretesting (n = 50).	13**

**Supplementary Table S12. Categorization results for weakly social norm violating vignettes (Mu et al., 2015): moral vs. conventional norm violation classification based on pretesting (n = 50).	18**

**Supplementary Table S13. Categorization results for strongly social norm violating vignettes (Mu et al., 2015): moral vs. conventional norm violation classification based on pretesting (n = 50).	21**

**Supplementary Table S14. Illustrative examples of direct and euphemized stimulus pairs.	24**

**Supplementary Figure S1. Model-based surprisal of critical words across experimental conditions.	25**

**Supplementary Figure S2. ERPs to non-euphemized, euphemized and control critical words with new preprocessing (Filtering in the 0.1–30 Hz). Significant differences of ERPs are indicated by red color.	26**

**Supplementary Figure S3. Grand average ERPs (Filtering in the 0.1–30 Hz) waveform in three experimental conditions: non- euphemized, euphemized and control critical words	26**

### **Supplementary Table S1.** The table of pairwise t-test for P600 to non-euphemized, euphemized and control critical words

|  | **Mean values** | **t-value** | **p-value** | **df** |
| --- | --- | --- | --- | --- |
| ***P600 mean amplitudes (500-700 ms)*** | | | | |
| **Non-euphemism - Euphemism** | 0.864 | 3.643 | <0.001 | 33 |
| **Non-euphemism - control** | 0.527 | 2.537 | 0.016 | 35 |
| **Euphemism - Control** | -0.532 | -2.875 | 0.006 | 34 |
| ***P600 max amplitudes (500-700 ms)*** |  |  |  |  |
| **Non-euphemism - Euphemism** | 0.614 | 2.491 | 0.018 | 31 |
| **Non-euphemism - control** | 0.684 | 2.392 | 0.022 | 36 |
| **Euphemism - Control** | -0.525 | -2.321 | 0.026 | 35 |

**Supplementary Table S2.** Stimuli in English (Translated from Russian)

| **id** | **Sentence before**  **stimuli (in russian)** | **Non-euphemized**  **stimuli (in russian)** | **Euphemized**  **stimuli (in russian)** | **Neutral stimuli (in russian)** | **Sentence before stimuli** | **Non-euphemized stimuli** | **Euphemized stimuli** | **Neutral stimuli** |
| --- | --- | --- | --- | --- | --- | --- | --- | --- |
| **1** | Артист выступал перед залом, полном жертв холокоста. | Своим выступлением он их оскорбил | Своим выступлением он их задел | Своим выступлением он их растрогал | The artist performed in front of a hall full of Holocaust victims. | By his performance, he could insult them. | By his performance, he could hurt their feelings. | By his performance, he could move them. |
| **2** | В зоне заражения находилось много граждан, важно было остановить вирус. | Власти прибегли к их расстрелу | Власти прибегли к их ликвидации | Власти прибегли к их лечению | There were many civilians in the contamination zone; it was important to stop the virus. | The authorities resorted to shooting them. | The authorities resorted to eliminating them. | The authorities resorted to treating them. |
| **3** | В многодетной семье было несколько приемных детей. | В целях воспитания родители прибегли к избиению | В целях воспитания родители прибегли к рукоприкладству | В целях воспитания родители прибегли к поощрению | A large family had several foster children. | For educational purposes, the parents resorted to beating them. | For educational purposes, the parents resorted to physical discipline. | For educational purposes, the parents resorted to encouragement. |
| **4** | В прошлом году власти провели реформу образования. | Это привело отрасль к новым кризисам | Это привело отрасль к новым вызовам | Это привело отрасль к новым возможностям | Last year, the authorities implemented an education reform. | This led the industry to a new crisis. | This led the industry to new challenges. | This led the industry to new opportunities. |
| **5** | В разгар эпидемии в больницах города не хватало врачей. | В это время многие из них были уволены | В это время многие из них были сокращены | В это время многие из них были премированы | During the peak of the epidemic, city hospitals lacked doctors. | At that time, many of them were fired. | At that time, many of them were laid off. | At that time, many of them were given bonuses. |
| **6** | Власти обещали сохранить количество учителей в сельских школах. | В последствии многие учителя были уволены | В последствии многие учителя были сокращены | В последствии многие учителя были премированы | The authorities promised to keep the number of teachers in rural schools. | Subsequently, many teachers were fired. | Subsequently, many teachers were laid off. | Subsequently, many teachers were given bonuses. |
| **7** | Военные приняли мирного жителя за террориста. | В процессе задержания они его убили | В процессе задержания они его ликвидировали | В процессе задержания они это поняли | The military mistook a civilian for a terrorist. | During the arrest, they killed him. | During the arrest, they neutralized him. | During the arrest, they realized their mistake. |
| **8** | Волонтер призывал граждан жертвовать пострадавшим от землетрясений. | Чтобы собрать больше, он прибегал к вранью | Чтобы собрать больше, он прибегал к неправде | Чтобы собрать больше, он прибегал к красноречию | A volunteer urged citizens to donate to earthquake victims. | To collect more, he resorted to lying. | To collect more, he resorted to exaggeration. | To collect more, he resorted to eloquence. |
| **9** | Враждующие кланы обещали гражданам прекратить размолвки. | На следующий день на улицах произошли масштабные битвы | На следующий день на улицах произошли масштабные столкновения | На следующий день на улицах произошли масштабные гуляния | The warring clans promised citizens to end their squabbles. | The next day, large-scale battles took place on the streets. | The next day, large-scale clashes took place on the streets. | The next day, large-scale festivities took place on the streets. |
| **10** | Врач спросил донора крови о его ВИЧ статусе. | Донор решил о нем наврать | Донор решил о нем утаить | Донор решил о нем рассказать | A doctor asked a blood donor about his HIV status. | The donor decided to lie about it. | The donor decided to conceal it. | The donor decided to disclose it. |
| **11** | Группа лиц хотела, чтобы их голос прозвучал во всеуслышанье. | Они устроили уличные беспорядки | Они устроили уличные шествия | Они устроили уличные концерты | A group of people wanted their voice to be heard loud and clear. | They staged a street riot. | They staged a street march. | They staged a street concert. |
| **12** | Дворовые мальчишки каждый вечер играли в футбол. | Проигравшего вратаря они избили | Проигравшего вратаря они поколачивали | Проигравшего вратаря они утешили | Local boys played football every evening. | They would beat the losing goalkeeper. | They would rough up the losing goalkeeper. | They would comfort the losing goalkeeper. |
| **13** | Дедушка пережил инфаркт и попросил домочадцев жить мирно. | В ответ на это они демонстрировали постоянные ссоры | В ответ на это они демонстрировали постоянные разногласия | В ответ на это они демонстрировали постоянные объятия | The grandfather survived a heart attack and asked his household to live in peace. | In response, they demonstrated constant quarrels. | In response, they demonstrated constant disagreements. | In response, they demonstrated constant hugs. |
| **14** | Директор банка должен был четко доложить вкладчикам о проблемах. | Он решил об этом наврать | Он решил об этом утаить | Он решил об этом рассказать | The bank director had to report the problems clearly to the depositors. | He decided to lie about it. | He decided to conceal it. | He decided to tell the truth. |
| **15** | Женщин в очереди попросили вести себя культурно. | Сразу после этого у них началась ссора | Сразу после этого у них начались разногласия | Сразу после этого у них началось затишье | The women in line were asked to behave civilly. | Immediately after, they started a quarrel. | Immediately after, they started a disagreement. | Immediately after, a lull set in. |
| **16** | Женщина стояла в длинной очереди и у нее было мало времени. | Через некоторое время она начала скандал | Через некоторое время она начала конфликт | Через некоторое время она начала напевать | A woman was standing in a long line and had little time. | After a while, she started a scandal. | After a while, she started a conflict. | After a while, she started humming. |
| **17** | Женщину в автобусе попросили немного подвинуться. | В ответ на это она начала скандал | В ответ на это она начала конфликт | В ответ на это она начала подвигаться | A woman on a bus was asked to move over a bit. | In response, she started a scandal. | In response, she started a conflict. | In response, she started to move over. |
| **18** | Инвалид приехал в модный магазин. | Один из продавцов-консультантов его оскорбил | Один из продавцов-консультантов его задел | Один из продавцов-консультантов его обслужил | A disabled person arrived at a fashionable store. | One of the sales assistants insulted him. | One of the sales assistants hurt his feelings. | One of the sales assistants served him. |
| **19** | Конкурирующие партии не согласились с результатами выборов. | Это привело их сторонников к серьезным сражениям | Это привело их сторонников к серьезным столкновениям | Это привело их сторонников к серьезным размышлениям | The competing parties disagreed with the election results. | This led their supporters to serious fighting. | This led their supporters to serious clashes. | This led their supporters to serious reflection. |
| **20** | Местные жители были против вырубки леса. | Они организовали уличные беспорядок. | Они организовали уличные шествие. | Они организовали уличные опрос. | Local residents were against deforestation. | They organized a street riot. | They organized a street march. | They organized a street poll. |
| **21** | Местные руководители ввели новый налог. | Это привело регион к новым экономическим кризисам | Это привело регион к новым экономическим вызовам | Это привело регион к новым экономическим успехам | Local officials introduced a new tax. | This led the region to a new economic crisis. | This led the region to new economic challenges. | This led the region to new economic success. |
| **22** | Мигранты встретились чтобы поделить сферы влияния. | Они закончили уличными сражениямм | Они закончили уличными столкновениями | Они закончили уличными гуляниями | Migrants met to divide spheres of influence. | They ended up with street fighting. | They ended up with street clashes. | They ended up with street revelry. |
| **23** | Миллиардер просил детей продолжить пожертвования после его смерти. | В дальнейшем они были уменьшены | В дальнейшем они были скорректированы | В дальнейшем они были сохранены | The billionaire asked the children to continue donations after his death. | Subsequently, they were reduced. | Subsequently, they were adjusted. | Subsequently, they were maintained. |
| **24** | Молодая семья никак не могла получить ипотеку. | Сотрудник банка предложил оформить ее за взятку | Сотрудник банка предложил оформить ее за подарок | Сотрудник банка предложил оформить ее позже | A young family could not get a mortgage. | The bank employee offered to arrange it for a bribe. | The bank employee offered to arrange it for a gift. | The bank employee offered to arrange it later. |
| **25** | Молодой министр финансов провел ряд реформ | Это привело накопления к обесцениванию | Это привело накопления к девальвации | Это привело накопления к увеличению | The young finance minister carried out a series of reforms. | This led to the devaluation of savings. | This led to the depreciation of savings. | This led to the increase of savings. |
| **26** | Молодой человек не набрал необходимые баллы для поступления. | Родители решили предложить ректору взятку | Родители решили предложить ректору подарок | Родители решили предложить ректору поговорить | The young man did not score the necessary points for admission. | The parents decided to offer the rector a bribe. | The parents decided to offer the rector a gift. | The parents decided to offer the rector a conversation. |
| **27** | Мужчина взял в жены молодую девушку. | Если ей не удавался ужин, он ее избивал | Если ей не удавался ужин, он ее поколачивал | Если ей не удавался ужин, он ее успокаивал | A man married a young girl. | If she failed at dinner, he would beat her. | If she failed at dinner, he would rough her up. | If she failed at dinner, he would calm her down. |
| **28** | Мужчина привез на дачу много пиротехники. | В результате этого его соседи услышали взрыв | В результате этого его соседи услышали хлопок | В результате этого его соседи услышали салют | A man brought a lot of fireworks to his dacha. | As a result, his neighbors heard an explosion. | As a result, his neighbors heard a pop. | As a result, his neighbors heard fireworks. |
| **29** | Муниципалитет обещали сохранить бюджет на поддержку приюта для животных. | Недавно они решили его уменьшить | Недавно они решили его скорректировать | Недавно они решили его увеличить | The municipality promised to maintain the budget for the animal shelter. | Recently, they decided to reduce it. | Recently, they decided to adjust it. | Recently, they decided to increase it. |
| **30** | На собеседовании мужчину спросили о навыках. | Он решил соглать | Он решил приукрасить | Он решил рассказать | At a job interview, the man was asked about his skills. | He decided to lie. | He decided to embellish. | He decided to tell the truth. |
| **31** | Научный коллектив получил финансовый грант. | В отчете по нему были выявлены признаки воровства | В отчете по нему были выявлены признаки хищения | В отчете по нему были выявлены признаки успеха | The research team received a financial grant. | In the report, signs of theft were found. | In the report, signs of embezzlement were found. | In the report, signs of success were found. |
| **32** | Подозреваемые в преступлении пытались скрыться. | Спецслужбы прибегли к убийству | Спецслужбы прибегли к ликвидации | Спецслужбы прибегли к переговорам | The crime suspects tried to escape. | The special services resorted to killing them. | The special services resorted to eliminating them. | The special services resorted to negotiations. |
| **33** | Руководители воюющих государств договорились о перемирии | Дальнейшие действия приграничных войск привели к битве | Дальнейшие действия приграничных войск привели к столкновению | Дальнейшие действия приграничных войск привели к перемирию | The leaders of the warring states agreed on a truce. | The subsequent actions of the border troops led to a battle. | The subsequent actions of the border troops led to a clash. | The subsequent actions of the border troops led to a truce. |
| **34** | Руководители нового государства хотели установить порядок и безопасность. | При совершении преступлений они прибегали к расстрелу | При совершении преступлений они прибегали к ликвидации | При совершении преступлений они прибегали к выговору | The leaders of the new state wanted to establish order and security. | When committing crimes, they resorted to shooting. | When committing crimes, they resorted to liquidation. | When committing crimes, they resorted to reprimands. |
| **35** | Руководитель докладывал об успехах компании. | В своем выступлении он допустил враньё | В своем выступлении он допустил неправду | В своем выступлении он допустил юмор | The director was reporting on the company's successes. | In his speech, he uttered lies. | In his speech, he uttered untruths. | In his speech, he uttered jokes. |
| **36** | С сотрудник получил доступ к бюджету организации. | В первые же дни он приступил к воровству | В первые же дни он приступил к хищению | В первые же дни он приступил к работе | An employee gained access to the organization's budget. | In the first few days, he started stealing. | In the first few days, he started embezzling. | In the first few days, he started working. |
| **37** | Свидетеля происшествия попросили рассказать все как было. | В ответ на это он решил солгать | В ответ на это он решил приукрасить | В ответ на это он решил рассказать | A witness to the incident was asked to tell it like it happened. | In response, he decided to lie. | In response, he decided to embellish. | In response, he decided to tell the truth. |
| **38** | Служба газа проводила ремонт аварийного оборудования. | Следствием их работы стал взрыв | Следствием их работы стал хлопок | Следствием их работы стал запуск | The gas service was repairing emergency equipment. | The result of their work was an explosion. | The result of their work was a pop. | The result of their work was a launch. |
| **39** | Финансовые службы должны были сохранить стабильность нацвалюты | Их дальнейшие действия привели к обесцениванию | Их дальнейшие действия привели к девальвации | Их дальнейшие действия привели к взлёту | The financial services were supposed to maintain the stability of the national currency. | Their subsequent actions led to devaluation. | Their subsequent actions led to depreciation. | Their subsequent actions led to a surge. |
| **40** | Циркачи дрессировали многих диких животных. | Для этого они практиковали избиение | Для этого они практиковали рукоприкладство | Для этого они практиковали поощрение | Circus trainers tamed many wild animals. | For this, they practiced beating them. | For this, they practiced physical force. | For this, they practiced encouragement. |

### **Supplementary Table S3.** Covariates Dictionary

| **Covariate** | **Description** | **Source / Tool** | **Scale / Unit** | **Notes** |
| --- | --- | --- | --- | --- |
| **Freq** | Word frequency (items per million) | [Russian spoken frequency dictionary](http://dict.ruslang.ru/freq.php?act=show&dic=freq_spoken&title=%D7%E0%F1%F2%EE%F2%ED%FB%E9%20%F1%EB%EE%E2%E0%F0%FC%20%E6%E8%E2%EE%E9%20%F3%F1%F2%ED%EE%E9%20%F0%E5%F7%E8) | ipm (items per million) | – |
| **valence_score** | Valence (positivity/negativity of the word) | RuSentiment Python library | Continuous score (higher = more positive) | – |
| **char_count** | Word length | Python: number of characters | Integer (characters) | – |
| **arousal** | Arousal (emotional intensity) | [NRC-VAD lexicon](https://saifmohammad.com/WebPages/nrc-vad.html) | Continuous score | – |
| **Mean_socialness** | Socialness of the word (social vs. non-social semantics) | [Bangor University resource](https://research.bangor.ac.uk/en/publications/quantifying-social-semantics-an-inclusive-definition-of-socialnes/) | Continuous score | 21 trials excluded due to missing values in the dictionary (N reduced to 3663) |

### **Supplementary Table S4.** Results for Linear Mixed-Effects Model with the LIKERT ratings as the dependent variable (“emot” – Non-euphemized, “less” – Euphemized and “filler” – Neutral).

| **Parameter** | **β** | **SE** | **z** | **p** | **95% CI** |
| --- | --- | --- | --- | --- | --- |
| **Intercept** | 3.373 | 0.110 | 30.554 | < .001 | [3.156, 3.589] |
| **C(condition)[T.emot]** | 0.425 | 0.048 | 8.847 | < .001 | [0.331, 0.519] |
| **C(condition)[T.filler]** | –2.755 | 0.048 | –57.317 | < .001 | [–2.849, –2.661] |
| **Freq** | –0.000 | 0.000 | –3.000 | 0.003 | [–0.001, –0.000] |
| **valence_score** | –0.246 | 0.042 | –5.889 | < .001 | [–0.328, –0.164] |
| **char_count** | 0.016 | 0.007 | 2.094 | 0.036 | [0.001, 0.030] |
| **arousal** | 0.103 | 0.044 | 2.336 | 0.020 | [0.017, 0.190] |
| **Group Var (random intercept)** | 0.193 | 0.041 | – | – | – |

### **Supplementary Table S5.** Results for Linear Mixed-Effects Model with the LIKERT ratings as the dependent variable (“emot” – Non-euphemized, “less” – Euphemized and “filler” – Neutral).

| **Parameter** | **β** | **SE** | **z** | **p** | **95% CI** |
| --- | --- | --- | --- | --- | --- |
| **Intercept** | 3.655 | 0.147 | 24.816 | < .001 | [3.366, 3.944] |
| **C(condition)[T.emot]** | 0.374 | 0.052 | 7.212 | < .001 | [0.273, 0.476] |
| **C(condition)[T.filler]** | –2.902 | 0.051 | –56.532 | < .001 | [–3.003, –2.802] |
| **Freq** | –0.000 | 0.000 | –2.869 | 0.004 | [–0.001, –0.000] |
| **valence_score** | –0.194 | 0.043 | –4.484 | < .001 | [–0.279, –0.109] |
| **char_count** | 0.016 | 0.008 | 1.888 | 0.059 | [–0.001, 0.032] |
| **Mean_socialness** | –0.024 | 0.017 | –1.431 | 0.152 | [–0.057, 0.009] |
| **Group Var (random intercept)** | 0.194 | 0.042 | – | – | – |

### **Supplementary Table S6.** Results for Linear Mixed-Effects Model with the P600 amplitude (μV) as the dependent variable (“emot” – Non-euphemized, “less” – Euphemized and “filler” – Neutral).

| **Parameter** | **β** | **SE** | **z** | **p** | **95% CI** |
| --- | --- | --- | --- | --- | --- |
| **Intercept** | 13.686 | 1.070 | 12.785 | < .001 | [11.588, 15.784] |
| **C(condition)[T.emot]** | 1.007 | 0.348 | 2.893 | 0.004 | [0.325, 1.689] |
| **C(condition)[T.filler]** | 0.496 | 0.348 | 1.426 | 0.154 | [–0.186, 1.178] |
| **Freq** | 0.001 | 0.001 | 0.771 | 0.441 | [–0.001, 0.003] |
| **valence_score** | –0.238 | 0.303 | –0.785 | 0.433 | [–0.831, 0.356] |
| **char_count** | –0.236 | 0.054 | –4.404 | < .001 | [–0.341, –0.131] |
| **arousal** | 0.017 | 0.320 | 0.055 | 0.956 | [–0.610, 0.645] |
| **Group Var (random intercept)** | 28.902 | 0.806 | – | – | – |

### **Supplementary Table S7.** Results for Linear Mixed-Effects Model with the P600 amplitude (μV) as the dependent variable (“emot” – Non-euphemized, “less” – Euphemized and “filler” – Neutral).

| **Parameter** | **β** | **SE** | **z** | **p** | **95% CI** |
| --- | --- | --- | --- | --- | --- |
| **Intercept** | 12.609 | 1.313 | 9.605 | < .001 | [10.036, 15.182] |
| **C(condition)[T.emot]** | 1.006 | 0.385 | 2.610 | 0.009 | [0.250, 1.762] |
| **C(condition)[T.filler]** | 0.535 | 0.381 | 1.403 | 0.161 | [–0.212, 1.282] |
| **Freq** | 0.001 | 0.001 | 0.614 | 0.539 | [–0.002, 0.003] |
| **valence_score** | –0.299 | 0.322 | –0.930 | 0.352 | [–0.931, 0.332] |
| **char_count** | –0.212 | 0.062 | –3.409 | 0.001 | [–0.333, –0.090] |
| **Mean_socialness** | 0.192 | 0.125 | 1.528 | 0.127 | [–0.054, 0.437] |
| **Group Var (random intercept)** | 30.183 | 0.843 | – | – | – |

### **Supplementary Table S8.** Descriptive statistics for word length.

| **condition** | **count** | **mean** | **std** |
| --- | --- | --- | --- |
| **non-euphemized** | 40 | 8,05 | 2,1 |
| **filler** | 40 | 8,98 | 2,49 |
| **euphemized** | 40 | 9,98 | 2,85 |

### **Supplementary Table S9.** Descriptive statistics for word frequency in literature.

| **condition** | **count** | **mean** | **std** |
| --- | --- | --- | --- |
| **non-euphemized** | 40 | 18,28 | 18,56 |
| **filler** | 40 | 101,59 | 196,19 |
| **euphemized** | 40 | 18,05 | 21,52 |

### **Supplementary Table S10**. Categorization results for non-euphemized vignettes: moral vs. conventional norm violation classification based on pretesting (n = 50).

| **vignette** | **factor score** | **ci_low_95** | **ci_high_95** | **bootstrap mean** | **moral/conventional evaluation** |
| --- | --- | --- | --- | --- | --- |
| Артист выступал перед залом, полном жертв холокоста. Своим выступлением он их оскорбил. | -0,519 | -1,882 | 1,97 | 0,572 | conventional |
| В зоне заражения находилось много граждан, важно было остановить вирус. Власти прибегли к их расстрелу. | -0,056 | -0,81 | 0,996 | 0,103 | conventional |
| В многодетной семье было несколько приемных детей. В целях воспитания родители прибегли к избиению. | -0,039 | -3,519 | 3,416 | -1,3 | moral |
| В прошлом году власти провели реформу образования. Это привело отрасль к новым кризисам | -0,315 | -1,07 | 1,267 | 0,296 | conventional |
| В разгар эпидемии в больницах города не хватало врачей. В это время многие из них были уволены. | -2,493 | -1,669 | 1,565 | -0,415 | moral |
| Власти обещали сохранить количество учителей в сельских школах. В последствии многие учителя были уволены. | 2,336 | -2,27 | 2,339 | 0,772 | conventional |
| Военные приняли мирного жителя за террориста. В процессе задержания они его убили. | 1,889 | -1,251 | 1,09 | -0,243 | moral |
| Волонтер призывал граждан жертвовать пострадавшим от землетрясений. Чтобы собрать больше, он прибегал к вранью | -0,395 | -2,399 | 2,503 | 0,874 | conventional |
| Враждующие кланы обещали гражданам прекратить размолвки. На следующий день на улицах произошли масштабные битвы. | -0,747 | -3,372 | 3,242 | -1,278 | moral |
| Врач спросил донора крови о его ВИЧ статусе. Донор решил о нем наврать. | -0,29 | -4,434 | 4,338 | -1,734 | moral |
| Группа лиц хотела, чтобы их голос прозвучал во всеуслышанье. Они устроили уличные беспорядки. | -1,624 | -2,107 | 2,238 | 0,726 | conventional |
| Дворовые мальчишки каждый вечер играли в футбол. Проигравшего вратаря они избивали. | -3,049 | -1,245 | 0,992 | -0,238 | moral |
| Дедушка пережил инфаркт и попросил домочадцев жить мирно. В ответ на это они демонстрировали постоянные ссоры. | -0,681 | -1,296 | 1,398 | 0,324 | conventional |
| Директор банка должен был четко доложить вкладчикам о проблемах. Он решил об этом наврать. | 0,743 | -2,463 | 2,582 | 0,838 | conventional |
| Женщин в очереди попросили вести себя культурно. Сразу после этого у них началась ссора. | 1,561 | -2,015 | 1,934 | -0,65 | moral |
| Женщина стояла в длинной очереди и у нее было мало времени. Через некоторое время она начала скандал. | -0,394 | -1,818 | 1,904 | 0,576 | conventional |
| Женщину в автобусе попросили немного подвинуться. В ответ на это она начала скандал. | -3,48 | -0,698 | 0,747 | 0,069 | conventional |
| Инвалид приехал в модный магазин. Один из продавцов-консультантов его оскорбил | 1,757 | -0,873 | 0,97 | 0,191 | conventional |
| Конкурирующие партии не согласились с результатами выборов. Это привело их сторонников к серьезным сражениям. | -0,018 | -1,444 | 1,304 | -0,413 | moral |
| Местные жители были против вырубки леса. Они организовали уличные беспорядки. | -2,53 | -2,684 | 2,816 | 0,983 | conventional |
| Местные руководители ввели новый налог. Это привело регион к новым экономическим кризисам. | 0,617 | -2,782 | 2,585 | -1,001 | moral |
| Мигранты встретились чтобы поделить сферы влияния. Они закончили уличными сражениями. | -3,619 | -1,769 | 1,915 | 0,525 | conventional |
| Миллиардер просил детей продолжить пожертвования после его смерти. В дальнейшем они были уменьшены. | 0,381 | -2,197 | 2,327 | 0,812 | conventional |
| Молодая семья никак не могла получить ипотеку. Сотрудник банка предложил оформить ее за взятку. | -3,35 | -1,268 | 1,215 | -0,286 | moral |
| Молодой министр финансов провел ряд реформ. Это привело накопления к обесцениванию. | 0,477 | -1,016 | 1,201 | 0,226 | conventional |
| Молодой человек не набрал необходимые баллы для поступления. Родители решили предложить ректору взятку. | 2,411 | -2,331 | 2,413 | 0,806 | conventional |
| Мужчина взял в жены молодую девушку. Если ей не удавался ужин, он ее избивал. | 1,233 | -1,414 | 1,22 | -0,345 | moral |
| Мужчина привез на дачу много пиротехники. В результате этого его соседи услышали взрыв. | -1,231 | -2,764 | 2,844 | 1,033 | conventional |
| Муниципалитет обещали сохранить бюджет на поддержку приюта для животных. Недавно они решили его уменьшить | -1,777 | -0,694 | 0,588 | -0,138 | moral |
| На собеседовании мужчину спросили о навыках. Он решил соглать. | -0,937 | -2,369 | 2,31 | -0,832 | moral |
| Научный коллектив получил финансовый грант. В отчете по нему были выявлены признаки воровства. | 2,109 | -3,893 | 3,771 | -1,504 | moral |
| Подозреваемые в преступлении пытались скрыться. Спецслужбы прибегли к убийству. | -0,821 | -0,688 | 0,71 | 0,045 | conventional |
| Руководители воюющих государств договорились о перемирии. Дальнейшие действия приграничных войск привели к битве. | 0,482 | -0,943 | 1,036 | 0,191 | conventional |
| Руководители нового государства хотели установить порядок и безопасность. При совершении преступлений они прибегали к расстрелу. | 2,11 | -2,018 | 2,159 | 0,663 | conventional |
| Руководитель докладывал об успехах компании. В своем выступлении он допустил враньё. | -2,8 | -2,04 | 1,866 | -0,674 | moral |
| Свидетеля происшествия попросили рассказать все как было. В ответ на это он решил солгать. | 1,798 | -1,422 | 1,545 | 0,391 | conventional |
| Служба газа проводила ремонт аварийного оборудования. Следствием их работы стал взрыв. | 1,836 | -1,651 | 1,773 | 0,49 | conventional |
| Сотрудник получил доступ к бюджету организации. В первые же дни он приступил к воровству. | -0,478 | -3,621 | 3,515 | -1,406 | moral |
| Финансовые службы должны были сохранить стабильность нацвалюты Их дальнейшие действия привели к обесцениванию. | 3,03 | -2,929 | 2,792 | -1,071 | moral |
| Циркачи дрессировали многих диких животных. Для этого они практиковали избиение. | 2,363 | -2,181 | 2,318 | 0,725 | conventional |

### **Supplementary Table S11.** Categorization results for euphemized vignettes: moral vs. conventional norm violation classification based on pretesting (n = 50).

| **vignette** | **factor score** | **ci_low_95** | **ci_high_95** | **bootstrap mean** | **moral/conventional evaluation** |
| --- | --- | --- | --- | --- | --- |
| Артист выступал перед залом, полном жертв холокоста. Своим выступлением он их задел. | -3,248 | -2,139 | 2,282 | 0,739 | conventional |
| В зоне заражения находилось много граждан, важно было остановить вирус. Власти прибегли к их ликвидации. | 1,227 | -0,865 | 0,798 | -0,14 | conventional |
| В многодетной семье было несколько приемных детей. В целях воспитания родители прибегли к рукоприкладству. | -2,059 | -3,903 | 3,824 | -1,509 | moral |
| В прошлом году власти провели реформу образования. Это привело отрасль к новым вызовам | -0,193 | -1,298 | 1,564 | 0,678 | conventional |
| В разгар эпидемии в больницах города не хватало врачей. В это время многие из них были сокращены. | -1,217 | -2,087 | 2,219 | 0,675 | conventional |
| Власти обещали сохранить количество учителей в сельских школах. В последствии многие учителя были сокращены. | -0,104 | -2,796 | 2,63 | -0,982 | moral |
| Военные приняли мирного жителя за террориста. В процессе задержания они его ликвидировали. | -0,891 | -1,829 | 1,715 | -0,506 | conventional |
| Волонтер призывал граждан жертвовать пострадавшим от землетрясений. Чтобы собрать больше, он прибегал к неправде | -0,395 | -2,399 | 2,503 | 0,874 | conventional |
| Враждующие кланы обещали гражданам прекратить размолвки. На следующий день на улицах произошли масштабные столкновения. | -1,467 | -2,541 | 2,419 | -0,943 | moral |
| Врач спросил донора крови о его ВИЧ статусе. Донор решил о нем утаить. | 3,11 | -3,81 | 3,691 | -1,449 | moral |
| Группа лиц хотела, чтобы их голос прозвучал во всеуслышанье. Они устроили уличные шествия. | 1,361 | -1,622 | 1,723 | 0,488 | conventional |
| Дворовые мальчишки каждый вечер играли в футбол. Проигравшего вратаря они поколачивали. | -1,344 | -1,982 | 1,778 | -0,591 | moral |
| Дедушка пережил инфаркт и попросил домочадцев жить мирно. В ответ на это они демонстрировали постоянные разногласия. | 2,948 | -2,175 | 2,047 | -0,719 | conventional |
| Директор банка должен был четко доложить вкладчикам о проблемах. Он решил об этом утаить. | 1,742 | -0,814 | 0,671 | -0,083 | conventional |
| Женщин в очереди попросили вести себя культурно. Сразу после этого у них началось разногласие. | -1,7 | -2,782 | 2,87 | 1,031 | moral |
| Женщина стояла в длинной очереди и у нее было мало времени. Через некоторое время она начала конфликт. | 1,977 | -2,193 | 2,263 | 0,78 | conventional |
| Женщину в автобусе попросили немного подвинуться. В ответ на это она начала конфликт. | 1,619 | -1,967 | 2,029 | 0,658 | conventional |
| Инвалид приехал в модный магазин. Один из продавцов-консультантов его задел | 1,757 | -0,873 | 0,97 | 0,191 | conventional |
| Конкурирующие партии не согласились с результатами выборов. Это привело их сторонников к серьезным столкновениям. | -0,124 | -0,693 | 0,518 | -0,048 | conventional |
| Местные жители были против вырубки леса. Они организовали уличные шествия. | 0,133 | -1,706 | 1,605 | -0,418 | moral |
| Местные руководители ввели новый налог. Это привело регион к новым экономическим вызовам. | 2,644 | -2,364 | 2,21 | -0,788 | conventional |
| Мигранты встретились чтобы поделить сферы влияния. Они закончили уличными столкновениями. | -1,567 | -3,507 | 3,417 | -1,361 | moral |
| Миллиардер просил детей продолжить пожертвования после его смерти. В дальнейшем они были скорректированы. | 2,479 | -2,071 | 2,266 | 0,749 | conventional |
| Молодая семья никак не могла получить ипотеку. Сотрудник банка предложил оформить ее за подарок. | -3,444 | -3,334 | 3,423 | 1,262 | conventional |
| Молодой министр финансов провел ряд реформ. Это привело накопления к девальвации. | 2,985 | -1,831 | 1,937 | 0,603 | moral |
| Молодой человек не набрал необходимые баллы для поступления. Родители решили предложить ректору подарок. | -3,473 | -1,501 | 1,31 | -0,423 | conventional |
| Мужчина взял в жены молодую девушку. Если ей не удавался ужин, он ее поколачивал. | -2,884 | -3,938 | 3,828 | -1,503 | conventional |
| Мужчина привез на дачу много пиротехники. В результате этого его соседи услышали хлопок. | -1,198 | -0,954 | 1,017 | 0,235 | moral |
| Муниципалитет обещали сохранить бюджет на поддержку приюта для животных. Недавно они решили его скорректировать | -1,777 | -0,694 | 0,588 | -0,138 | moral |
| На собеседовании мужчину спросили о навыках. Он решил приукрасить. | 2,5 | -2,337 | 2,231 | -0,792 | moral |
| Научный коллектив получил финансовый грант. В отчете по нему были выявлены признаки хищения. | 0,948 | -3,658 | 3,569 | -1,385 | moral |
| Подозреваемые в преступлении пытались скрыться. Спецслужбы прибегли к ликвидации. | -0,172 | -2,78 | 2,95 | 1,027 | conventional |
| Руководители воюющих государств договорились о перемирии. Дальнейшие действия приграничных войск привели к столкновению. | -3,093 | -3,516 | 3,414 | -1,328 | conventional |
| Руководители нового государства хотели установить порядок и безопасность. При совершении преступлений они прибегали к ликвидации. | 3,594 | -3,858 | 3,727 | -1,455 | conventional |
| Руководитель докладывал об успехах компании. В своем выступлении он допустил неправду. | 1,227 | -2,351 | 2,411 | 0,859 | moral |
| Свидетеля происшествия попросили рассказать все как было. В ответ на это он решил приукрасить. | -2,13 | -1,65 | 1,767 | 0,514 | conventional |
| Служба газа проводила ремонт аварийного оборудования. Следствием их работы стал хлопок. | 0,828 | -3,199 | 3,073 | -1,184 | conventional |
| Сотрудник получил доступ к бюджету организации. В первые же дни он приступил к хищению. | -2,33 | -1,358 | 1,452 | 0,344 | moral |
| Финансовые службы должны были сохранить стабильность нацвалюты Их дальнейшие действия привели к девальвации. | 1,548 | -2,051 | 1,865 | -0,599 | moral |
| Циркачи дрессировали многих диких животных. Для этого они практиковали рукоприкладство. | -1,414 | -1,72 | 1,773 | 0,558 | conventional |

### **Supplementary Table S12.** Categorization results for weakly social norm violating vignettes (Mu et al., 2015): moral vs. conventional norm violation classification based on pretesting (n = 50).

| **original vignette** | **vignette in russian** | **factor score** | **ci_low_95** | **ci_high_95** | **bootstrap mean** | **moral/conventional evaluation** |
| --- | --- | --- | --- | --- | --- | --- |
| Wang Yong is eating pancakes at the class. | Александр ест блинчики на уроке. | 2,035 | -0,78 | 0,674 | -0,067 | мораль |
| Li Xia is changing her baby’s diapers at the hotel lobby. | Алина меняет подгузник своему малышу в холле отеля. | -0,987 | -2,408 | 2,409 | -0,804 | moral |
| Li Yan is taking photos in the funeral. | Анастасия фотографирует на похоронах. | 2,899 | -3,843 | 3,693 | -1,454 | moral |
| Zhang Lei is cycling on the highway. | Анна катается на велосипеде по шоссе. | 0,61 | -1,297 | 1,12 | -0,328 | moral |
| Li Qiang is playing guitar in the hospital. | Артём играет на гитаре в больнице. | 0,976 | -2,635 | 2,537 | -0,889 | moral |
| Wang Jun is cursing at the family gathering. | Борис ругается на семейном собрании. | -0,901 | -0,863 | 1,078 | 0,192 | conventional |
| Wang Fang is chatting at the mediation class. | Вероника болтает на занятии по медиации. | -3,569 | -2,595 | 2,672 | 0,971 | conventional |
| Wang Fang is painting in the hospital. | Виктория рисует в больнице. | 1,742 | -1,662 | 1,781 | 0,488 | conventional |
| Li Xia is crying loud in the class. | Владимир громко плачет на уроке. | 1,749 | -0,763 | 0,901 | 0,057 | conventional |
| Li Mei is reading books in the church. | Галина читает книги в церкви. | -0,97 | -0,88 | 0,819 | -0,051 | moral |
| Wang Lei is listening to MP3 in the class. | Глеб слушает музыку через MP3-плеер на уроке. | -2,365 | -0,84 | 0,697 | -0,183 | moral |
| Zhang Tao is stretching arms when listening to the lecture. | Дарья потягивается во время лекции. | 0,879 | -3,236 | 3,131 | -1,212 | moral |
| Zhang Lei is taking on cell phone at the theater. | Дмитрий разговаривает по мобильному телефону в театре. | -0,694 | -0,776 | 0,623 | -0,096 | moral |
| Liu Fang is breastfeeding in the library. | Ева кормит грудью в библиотеке. | -1,904 | -3,806 | 3,923 | 1,562 | conventional |
| Li Jie is flirting in the doctor’s office. | Екатерина флиртует в кабинете врача. | 1,567 | -2,514 | 2,431 | -0,91 | moral |
| Wang Juan is holding hands with her boyfriend at the business meeting. | Елена держится за руки со своим парнем на деловой встрече. | -1,848 | -0,606 | 0,519 | -0,026 | moral |
| Wang qiang is praying in the choir performance. | Елизавета молится во время выступления хора. | -1,861 | -2,205 | 2,362 | 0,792 | conventional |
| Wang Xiuying is sunbathing in the funeral. | Зоя загорает на похоронах. | 2,097 | -3,114 | 2,971 | -1,184 | moral |
| Zhang Jie is laughing out loud in the funeral. | Илья громко смеётся на похоронах. | 1,463 | -2,581 | 2,68 | 0,972 | conventional |
| Zhang Li is brushing teeth in the hotel. | Кристина чистит зубы в отеле. | -0,039 | -0,78 | 0,825 | 0,021 | conventional |
| Li Jing is texting in the church. | Ксения пишет сообщения в церкви. | -0,196 | -2,511 | 2,598 | 0,877 | conventional |
| Li Wei is yawning with the boss in the meeting room. | Лев зевает с боссом в переговорной. | 1,866 | -1,941 | 1,824 | -0,605 | moral |
| Zhang Wei is yelling in the library. | Максим кричит в библиотеке. | 2,556 | -3,305 | 3,389 | 1,274 | conventional |
| Zhang Yan is dancing in the art museum. | Мария танцует в художественном музее. | 3,694 | -3,115 | 3,186 | 1,106 | conventional |
| Wang Juan is singing at the hospital. | Милана поёт в больнице. | 0,217 | -1,841 | 1,737 | -0,52 | moral |
| Wang qiang is joking with the boss in the meeting room. | Михаил шутит с боссом в переговорной. | 3,603 | -3,287 | 3,46 | 1,254 | conventional |
| Li Na is putting on lipstick in the church. | Надежда красит губы в церкви. | 1,924 | -1,66 | 1,519 | -0,502 | moral |
| Wang Tao is arguing in the elevator. | Никита спорит в лифте. | -3,161 | -0,583 | 0,543 | -0,047 | moral |
| Li Ming is hugging with his girlfriend in the meeting room. | Олег обнимается со своей девушкой в переговорной. | 0,262 | -1,439 | 1,35 | -0,369 | moral |
| Wang Xiulan is kissing in the doctor’s office. | Ольга целуется в кабинете врача. | -0,208 | -1,557 | 1,364 | -0,426 | moral |
| Liu Wei is chewing gum when attending the business negotiation. | Павел жуёт жвачку на деловых переговорах. | -1,015 | -2,239 | 2,212 | -0,747 | moral |
| Li Li is bargaining in the church. | Полина торгуется в церкви. | -2,247 | -3,236 | 3,361 | 1,317 | conventional |
| Wang Xiulan is sleeping at the class. | Светлана спит на уроке. | 1,435 | -1,073 | 0,927 | -0,28 | moral |
| Li Fang is clapping hands in the funeral. | Софья хлопает в ладоши на похоронах. | -0,943 | -2,824 | 2,799 | -1,042 | moral |

### **Supplementary Table S13.** Categorization results for strongly social norm violating vignettes (Mu et al., 2015): moral vs. conventional norm violation classification based on pretesting (n = 50).

| **original vignette** | **vignette in russian** | **factor score** | **ci_low_95** | **ci_high_95** | **bootstrap mean** | **moral/conventional evaluation** |
| --- | --- | --- | --- | --- | --- | --- |
| Wang Yong is eating pancakes on the bus. | Александр ест блинчики в автобусе. | -1,235 | -2,308 | 2,215 | -0,731 | moral |
| Li Xia is changing her baby’s diapers in the park. | Алина меняет подгузник своему малышу в парке. | -2,167 | -0,799 | 0,726 | -0,115 | moral |
| Li Yan is taking photos at the class. | Анастасия фотографирует на уроке. | -4,113 | -0,701 | 0,632 | -0,04 | moral |
| Zhang Lei is cycling at the pedestrians. | Анна катается на велосипеде среди пешеходов. | 2,134 | -3,108 | 3,178 | 1,202 | conventional |
| Li Qiang is playing guitar on the metro platform. | Артём играет на гитаре на платформе метро. | 3,105 | -1,415 | 1,292 | -0,327 | moral |
| Wang Jun is cursing in the park. | Борис ругается в парке. | 0,776 | -0,656 | 0,662 | -0,015 | moral |
| Wang Fang is chatting at the theater. | Вероника болтает в театре. | 1,309 | -2,339 | 2,241 | -0,856 | moral |
| Wang Fang is painting at the street corner. | Виктория рисует на уличной стене. | -0,83 | -1,835 | 1,668 | -0,512 | moral |
| Li Xia is crying loud on the bus. | Владимир громко плачет в автобусе. | 0,814 | -1,96 | 2,152 | 0,68 | conventional |
| Li Mei is reading books in the supermarket. | Галина читает книги в супермаркете. | -1,049 | -3,838 | 4,028 | 1,521 | conventional |
| Wang Lei is listening to MP3 at the bar. | Глеб слушает музыку через MP3-плеер в баре. | -1,31 | -1,507 | 1,392 | -0,354 | moral |
| Zhang Tao is stretching arms in the restaurant. | Дарья потягивается в ресторане. | 2,016 | -1,792 | 1,705 | -0,502 | moral |
| Zhang Lei is taking on cell phone at the post office. | Дмитрий разговаривает по мобильному телефону на почте. | 0,559 | -0,838 | 0,972 | 0,169 | conventional |
| Liu Fang is breastfeeding in the park. | Ева кормит грудью в парке. | -3,345 | -0,722 | 0,869 | 0,051 | conventional |
| Li Jie is flirting at the post office. | Екатерина флиртует на почте. | -1,054 | -3,263 | 3,395 | 1,232 | conventional |
| Wang Juan is holding hands with her boyfriend at the bank. | Елена держится за руки со своим парнем в банке. | 1,618 | -2,917 | 3,003 | 1,075 | conventional |
| Wang qiang is praying in the park. | Елизавета молится в парке. | 0,104 | -1,116 | 1,26 | 0,269 | conventional |
| Wang Xiuying is sunbathing in the campus. | Зоя загорает в кампусе. | -1,576 | -2,474 | 2,568 | 0,891 | conventional |
| Zhang Jie is laughing out loud on the bus. | Илья громко смеётся в автобусе. | -0,187 | -2,126 | 2,029 | -0,668 | moral |
| Zhang Li is brushing teeth in the public lavatory. | Кристина чистит зубы в общественном туалете. | -1,387 | -1,969 | 2,09 | 0,657 | conventional |
| Li Jing is texting in the gym. | Ксения пишет сообщения в спортзале. | -0,581 | -1,3 | 1,164 | -0,267 | moral |
| Li Wei is yawning when listening to the lecture. | Лев зевает во время лекции. | 1,601 | -4,677 | 4,797 | 1,882 | conventional |
| Zhang Wei is yelling on the metro. | Максим кричит в метро. | -1,99 | -1,149 | 1,195 | 0,299 | conventional |
| Zhang Yan is dancing at the subway platform. | Мария танцует на платформе метро. | 0,413 | -1,328 | 1,446 | 0,366 | conventional |
| Wang Juan is singing on the pedestrians. | Милана поёт среди пешеходов. | -0,755 | -3,796 | 3,854 | 1,508 | conventional |
| Wang qiang is joking with strangers in the airplane. | Михаил шутит с незнакомцами в самолёте. | -0,209 | -2,48 | 2,609 | 0,836 | conventional |
| Li Na is putting on lipstick at the bank. | Надежда красит губы в банке. | -2,018 | -1,857 | 1,811 | -0,541 | moral |
| Wang Tao is arguing in the park. | Никита спорит в парке. | 1,397 | -0,757 | 0,703 | -0,054 | moral |
| Li Ming is hugging with his girlfriend at the bank. | Олег обнимается со своей девушкой в банке. | 4,447 | -1,391 | 1,348 | -0,365 | moral |
| Wang Xiulan is kissing on the bus. | Ольга целуется в автобусе. | 3,069 | -2,164 | 2,214 | 0,746 | conventional |
| Liu Wei is chewing gum when attending the awards ceremony. | Павел жуёт жвачку на церемонии вручения наград. | -3,57 | -1,939 | 2,071 | 0,64 | conventional |
| Li Li is bargaining in the taxi. | Полина торгуется в такси. | 2,339 | -1,453 | 1,373 | -0,378 | moral |
| Wang Xiulan is sleeping on the bench in the park. | Светлана спит на скамейке в парке. | -2,77 | -1,002 | 0,886 | -0,176 | moral |
| Li Fang is clapping hands in the hotel lobby. | Софья хлопает в ладоши в холле отеля. | 1,145 | -1,412 | 1,634 | 0,409 | conventional |

### **Supplementary Table S14. Illustrative examples of direct and euphemized stimulus pairs.**

**Note.** The examples illustrate the semantic overlap between direct and euphemized critical words. Euphemized expressions were selected to preserve the same general event domain as their direct counterparts while reducing explicitness and evaluative directness in the vignette context. Definitions were adapted from Ozhegov and Shvedova’s *Explanatory Dictionary of the Russian Language* and the *Comprehensive Explanatory Dictionary of the Russian Language* edited by S. A. Kuznetsov.

| **Russian version/ English translation (for illustrative purposes only)** | **Definition from dictionary** | **Euphemism:**  **Russian version/ English translation (for illustrative purposes only)** | **Definition from dictionary** |
| --- | --- | --- | --- |
| оскорбить  to insult | to seriously **offend**, deeply humiliate, or injure a person’s honor and dignity by word or deed. | задеть  to hurt | t**o offend someone** or affect someone’s feelings; to touch or brush against someone or something while moving; |
| избиение  beating | **the infliction of blows** in the absence of clear resistance from the opponent | рукоприкладство  physical discipline | behavior or actions characterized by **the infliction of blows** |

**Supplementary Figure S1. Model-based surprisal of critical words across experimental conditions.** Surprisal was calculated as −log₂ P(word | preceding context) using ai-forever/rugpt3medium_based_on_gpt2 and is expressed in bits. Gray dots represent individual stimuli; gray lines connect matched stimulus triplets across conditions. Pairwise comparisons: all *p* > .2

### **Supplementary Figure S2.** ERPs to non-euphemized, euphemized and control critical words with new preprocessing (Filtering in the 0.1–30 Hz). Significant differences of ERPs are indicated by red color.

### **Supplementary Figure S3.** Grand average ERPs (Filtering in the 0.1–30 Hz) waveform in three experimental conditions: non- euphemized, euphemized and control critical words
